# Diversity on a small scale: phylogeography of the locally endemic dwarf succulent genus *Oophytum* (Aizoaceae) in the Knersvlakte of South Africa

**DOI:** 10.1093/aob/mcae207

**Published:** 2024-12-04

**Authors:** Sabrina A Schmidt, Ute Schmiedel, Frederic Carstens, Anna-Lena Rau, Barbara Rudolph-Bartsch

**Affiliations:** Institute of Plant Science and Microbiology, University of Hamburg, Hamburg, Germany; Institute of Plant Science and Microbiology, University of Hamburg, Hamburg, Germany; Institute of Plant Science and Microbiology, University of Hamburg, Hamburg, Germany; Institute of Plant Science and Microbiology, University of Hamburg, Hamburg, Germany; Centre of Methods, Leuphana University Lüneburg, Lüneburg, Germany; Institute of Plant Science and Microbiology, University of Hamburg, Hamburg, Germany

**Keywords:** Edaphism, habitat heterogeneity, Succulent Karoo Biome, AFLP, haplotype, comparative heatmaps, quartz habitat islands, geographical segregation, phylogeny, isolation barriers, palaeoclimatic glaciation, Pleistocene

## Abstract

**Background and Aims:**

*Oophytum* (Aizoaceae) is a locally endemic genus of the extremely fast-evolving subfamily Ruschioideae and consists of only two formally accepted species (*Oophytum nanum* and *Oophytum oviforme*). Both species are leaf-succulent dwarf shrubs and habitat specialists on quartz fields in the Knersvlakte, a renowned biodiversity hotspot in the arid winter-rainfall Succulent Karoo Biome of South Africa. Quartz fields present specialised patchy habitats with an island-like distribution in the landscape. *Oophytum oviforme* grows in the south-western part, whereas *O. nanum* covers most of the remaining Knersvlakte. These species co-occur in a small area, but within different quartz islands. We investigated the effects of the patchy distribution, environmental conditions and potential effects of palaeoclimatic changes on the genetics of *Oophytum*.

**Methods:**

Phylogenetic and population genetic analyses of 35 populations of the genus, covering its entire distribution area, were conducted using four chloroplast DNA markers and an amplified fragment length polymorphism dataset. These were combined with environmental data via a principal component analysis and comparative heatmap analyses.

**Key Results:**

The genetic pattern of the *Oophytum* metapopulation is a tripartite division, with northern, central and western groups. This geographical pattern does not correspond to the two-species concept of *Oophytum*. Only the western *O. oviforme* populations form a monophyletic lineage, whereas the central populations of *O. oviforme* are genetic hybrids of *O. nanum* populations. The highly restricted gene flow often resulted in private gene pools with very low genetic diversity, in contrast to the hybrid gene pools of the central and edge populations.

**Conclusions:**

*Oophytum* is an exceptional example of an extremely fast-evolving genus that illustrates the high speciation rate of the Ruschioideae and their success as one of the leading plant groups of the drought-prone Succulent Karoo Biome. The survival strategy of these dwarf quartz-field endemics is an interplay of adaptation to diverse island habitats, highly restricted gene flow, occasional long-distance dispersal, migration, founder effects and hybridisation events within a small and restricted area caused by glacial and interglacial changing climate conditions from the Pleistocene to the Present. These findings have important implications for future conservation management strategies.

## INTRODUCTION

Palaeoclimatic changes have often induced adaptations and migrations of populations, leading to accelerated evolutionary processes (Schnitzler *et al.*, 2012). Three important palaeoclimatic events have caused dramatic changes in south-western Africa (Cowling *et al.*, 2009). The development of the Great Escarpment in South Africa in the late Jurassic/early Cretaceous (Clark *et al.*, 2011), the onset of the Benguela Current and the Benguela Upwelling System (Dupont *et al.*, 2011), caused by glaciation of Antarctica in the Late Miocene, resulted in strong aridification of the region (Hoetzel *et al.*, 2015). This was the pivotal factor for the establishment of the winter-rainfall climate along the South African west coast, leading to the radiation of the species-rich Fynbos and Succulent Karoo Biomes (~10 Mya; Dupont *et al.*, 2011). Subsequent recurrent glaciations during the Pleistocene have repeatedly shifted these two biomes in a north–south direction up to now (Midgley and Roberts, 2001; Midgley *et al.*, 2010).

Today, the Succulent Karoo and the Fynbos, the two winter-rainfall biomes of southern Africa, are renowned for their high biodiversity (Mucina and Rutherford, 2006; Kreft and Jetz, 2007; Linder *et al.*, 2010). The semi-arid Succulent Karoo houses 6350 plant species, of which ~1000 are endemic to this biome (Driver *et al.*, 2003), thus making it the only arid biodiversity hotspot worldwide (Myers *et al.*, 2000). The low but highly predictable winter rainfall has been identified as one of the main drivers of the exceptionally high species richness in the Succulent Karoo (Hoffman and Cowling, 1987; Desmet, 2007). It provides an ameliorated temperature regime, rare occurrence of frost events, high humidity at night and frequent precipitation of dew, in addition to fog (von Willert *et al.*, 1992; Cowling *et al.*, 1998). These conditions favour the high frequency of leaf succulence with limited water storage capacity, Crassulacean acid metabolism (CAM), shallow root systems (von Willert *et al.*, 1992) and plant minutism (Midgley and Van Der Heyden, 1999). In addition to some long-lived species among the leaf-succulent dwarf shrubs that have been recorded to reach >50 years (Stopp, 1954), short- to medium-lived shrub life forms that reach 3–10 years are also common (Jürgens *et al.*, 1999). Most of the leaf-succulent plants with CAM are intolerant of occasional severe and long droughts (von Willert *et al.*, 1985), which enhances repeated diebacks of populations. The reliable winter rainfall, in turn, promotes regular germination of perennials and regrowth of populations, resulting in a high population turnover (Cowling *et al.*, 1998), which enhances the chances for speciation (Rosenzweig, 1995).

The ice plants (Aizoaceae) are one of the dominating and most species-rich plant families in the Succulent Karoo (Snijman, 2013). Dated phylogenetic trees of the Aizoaceae family suggest that the onset of diversification falls into a phase of deteriorating climate, opening an arid winter-rainfall mega-niche ~5–10 Mya (Klak *et al.*, 2004; Valente *et al.*, 2014; Cunha Neto *et al.*, 2024). Morphological key innovations of the ice plant family (wide-band tracheids and highly succulent leaves) might have been preconditions for the unprecedented rate at which the ice plants radiated successfully during that period (Landrum, 2001). A remarkably high habitat heterogeneity, driven by topography, geology and soil conditions of the Succulent Karoo (Petersen *et al.*, 2010), might have enhanced small-scale turnover and the species richness in the area (Schmiedel *et al.*, 2015; Eibes *et al.*, 2021).

Edaphically driven special habitats, such as the quartz fields or quartz habitat islands, house a high number of habitat specialists and local endemics (Schmiedel and Jürgens, 1999; Powell *et al.*, 2019; Eibes *et al.*, 2022). Quartz field habitats occur in six different regions of southern Africa, from the Fynbos Biome in the southern Cape (Curtis *et al.*, 2013) to Nama-Karoo Biome in southern Namibia, but occur most frequently in the Succulent Karoo Biome (Schmiedel, 2004). The lineages from which the quartz field floras in the different regions emerged are genetically distinct (Zhigila *et al.*, 2024) but evolved convergent traits leading to their adaptation to the similar ranges of habitat conditions on the quartz field, resulting in distinct floras of striking morphological similarity (Schmiedel, 2004). Throughout the subcontinent, the quartz field-specialised taxa are characterised by dwarf, compact growth forms with highly succulent leaves (Schmiedel and Jürgens, 1999; Schmiedel, 2004; Eibes *et al.*, 2022).

So far, 67 obligate quartz field plant taxa have been recorded in the Knersvlakte, of which 63 taxa (94 %) and two to three genera (depending on the taxonomic view) of the Aizoaceae family (*Argyroderma* N.E.Br., *Dactylopsis* N.E.Br. and *Oophytum* N.E.Br.) are locally endemic (Schmiedel, 2004).

Due to high salinity or shallow-stony soils with low soil pH, quartz fields are classified as edaphically arid special habitats (Schmiedel and Jürgens, 1999). As an adaptation to the harsh environmental conditions, quartz field specialists are slow growing and poor competitors. These features limit their competitive advantage to the adverse quartz field habitats.

**Fig. 1.**
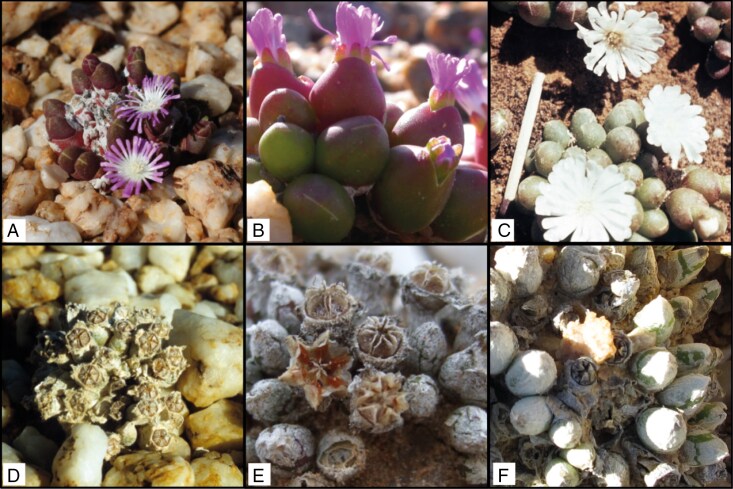
(A–C) Flowering plants of *Oophytum nanum* (A), *Oophytum oviforme* (B) and ‘*Oophytum nordenstamii*’ (C) (photograph by B. Nordenstam, taken at the type locality in 1962; photograph provided by the Herbarium Hamburgense from the inheritance of Professor Ihlenfeldt). (D–F) Plants with fruit capsules of *O. nanum* (D), *O. oviforme* (E) and ‘*O. nordenstamii*’ (F).

Quartz field habitats have a clearly demarcated, island-like distribution in the landscape, which is reflected in the patchy distribution of the habitat-specialised plant populations (Schmiedel *et al.*, 2015; Eibes *et al.*, 2022). The resulting spatial isolation of plant populations is likely to result in distinct population genetic patterns and radiation. Ellis *et al.* (2006) identified habitat specialisation as a key role in species radiation within another Knersvlakte-endemic quartz field genus, *Argyroderma* N.E.Br. Recent population genetic studies suggest species-specific patterns of population heterogeneity, which might be driven by respective dispersal modes (Musker *et al.*, 2020) or pollinator specialisation (Powell *et al.*, 2019). These studies suggest a high genetic diversity at different spatial scales, but the evolutionary processes and environmental drivers behind these patterns are still poorly understood. Sister species, which are analysed in comparison, are particularly suitable for such investigations (Ellis *et al.*, 2014). For a comprehensive and comparative phylogeographical study, we selected the locally endemic and obligate quartz field dwelling genus *Oophytum* (Aizoaceae) from the Knersvlakte (Fig. 1), because this genus, with two species, represents a perfect study object for this purpose. A phylogenetic study of the most diversified subtribe Ruschieae by Klak *et al.* (2013) placed *Oophytum* within the *Dicrocaulon* clade together with the genera *Dicrocaulon* N.E.Br., *Diplosoma* Schwantes and *Monilaria* Schwantes. These molecular results differed from the previous morphological study by Hartmann (1998), which placed *Oophytum* in the *Mitrophyllum*-Group together with the genera *Dicrocaulon*, *Diplosoma*, *Disphyma* N.E.Br. *Glottiphyllum* Haw. ex N.E.Br., *Jacobsenia* L.Bolus & Schwantes, *Meyerophytum* Schwantes, *Mitrophyllum* Schwantes and *Monilaria*. Most of these genera are compact leaf-succulent dwarf shrubs and are adapted to the prevailing harsh conditions of the quartz fields. With the herein presented work, we are building on the taxonomic revision of *Oophytum* by Ihlenfeldt (1978).

**Fig. 2.**
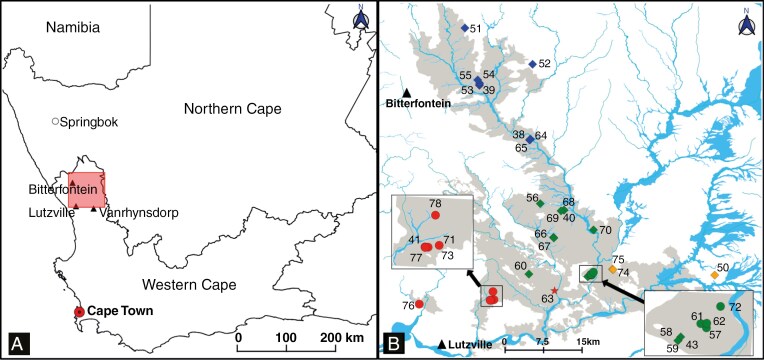
(A) Overview map of the two western Provinces of South Africa, with distribution range of *Oophytum* in the Knersvlakte (red framed square). (B) Detailed distribution map of 35 *Oophytum* populations created with QGIS v.3.22.0 and VEGMAP2018 shapefiles (https://www.sanbi.org/biodiversity/foundations/national-vegetation-map/) (ovals = *O. oviforme* populations, hexagon = *O. nanum* populations, star = ‘*O. nordenstamii*’ population, red = west, green = centre, blue = north, orange = east, numbers beside symbols are the population numbers, grey areas = SKk 3 Knersvlakte Quartz Vygieveld, blue lines = AZi 1 Namaqualand Riviere).

The genus currently consists of only two taxonomically defined species with an uneven distribution pattern (see Fig. 2). *Oophytum oviforme* (N.E.Br.) N.E.Br. is largely restricted to the south-west of the Knersvlakte, whereas the distribution of *O. nanum* (Schltr.) L.Bolus is much broader and stretches from the centre to the far north of the Knersvlakte. In the southern part of the Knersvlakte the two species co-occur, but their populations are found on different quartz field habitats (Ihlenfeldt, 1978). Bolus (1962) described a third species, *Oophytum nordenstamii* L.Bolus, which consisted of one population only and was located geographically between the western *O. oviforme* and the central *O. nanum* populations (Fig. 2). *Oophytum nordenstamii* was subsequently included in *O. oviforme* by Ihlenfeldt (1978).

Due to the restriction of the taxa to specific quartz field habitats, the *Oophytum* populations have a patchy distribution of clearly delineated, dense populations in the landscape. The spatial patterns of distribution make *Oophytum* an ideal system to study phylogeographical relationships and their evolution in relation to their potential environmental drivers. To analyse the genus in more detail, we selected two different genomic marker systems, which provide different resolutions within the entire genus. Four different and frequently used chloroplast markers (*mat*K, *trn*L-*trn*F, *trn*Q-*rps*16 and *trn*S-*trn*G), which have already been used in phylogenetic studies of Aizoaceae by Klak *et al.* (2013), should reveal the phylogenetic relationship of the sister species and their position within the *Dicrocaulon* clade. Additionally, an amplified fragment length polymorphism (AFLP) fingerprinting was chosen for a higher infraspecific resolution to examine gene flow within the metapopulation of the genus throughout the distribution range. Furthermore, we compared the genetic patterns to the geographical distribution with environmental characteristics of their habitats and population-specific traits in combined analyses. These results were then linked to palaeoclimatic changes.

With this approach, we aim to answer the following questions:

(1) Are the two taxonomically defined species genetically distinct?
(2) Can we find signs of hybridisation between the two species where they co-occur?
(3) Do the population genetic patterns reflect the spatial distribution of the quartz fields?
(4) Can we identify environmental drivers that influence diversification within the genus?

## MATERIALS AND METHODS

### Study taxa

The genus *Oophytum*, with its two currently accepted species, *O. nanum* and *O. oviforme*, belongs to the most species-rich and most rapidly evolving subfamily of the Aizoaceae, the Ruschioideae. Both species are compact leaf-succulent dwarf shrubs with small, completely connate, egg-shaped or nearly spherical corpuscles (Fig. 1; Table 1). The morphological differences between the two species are not always apparent, but *O. nanum* can be distinguished from *O. oviforme* by the visible number of leaf pairs and the keeling of the leaves. Both species flower during the rainy season, with about two rows of numerous magenta-coloured petaloid staminodes and with a white base as a visible perianth (Ihlenfeldt, 1978). White flowers have been reported for the former ‘*O. nordenstamii*’ population (Bolus, 1962; Ihlenfeldt, 1978). Genetic exchange is achieved through insect pollination. Hymenoptera, Diptera (bee flies), Coleoptera (monkey beetles) and Apoidea (bees) have been reported as frequent visitors of Aizoaceae (Struck, 1994, 1995; Mayer *et al.*, 2006; Mayer and Pufal, 2007). The fruits are hygrochastic capsules with a diameter of 3.8 mm (*O. nanum*) to 4.8 mm (*O. oviforme*) on average (Ihlenfeldt, 1978). They open after rain events, and seeds are ejected by splashing raindrops (Ihlenfeldt, 1960; Parolin, 2006). Thus, primary dispersal is over short distances (mainly <1 m; Parolin, 2001). The seeds germinate after 3–6 days (Ihlenfeldt, 1978). Secondary dispersal events of the small seeds are facilitated by surface run-off during heavy rainfall (medium-distance dispersal, downslope) and by frequently occurring dust storms (long-distance dispersal; Powell *et al.*, 2019, 2022). Given that this genus reproduces exclusively by sexual reproduction, successful seed dispersal is of central importance.

**Table 1.** Morphological characters of *Oophytum* (taken from Ihlenfeldt, 1978).

	*Oophytum nanum*	*Oophytum oviforme*
Leaves	Two (or three) leaf pairs (= corpuscles) per growing season are formed; both are visible	Two leaf pairs per growing season are formed, but only the first is visible. The second is hidden inside
	Both leaf pairs are succulent	Second leaf pair is succulent in the upper part and membranous dry at the bottom
	Leaves are keeled	Leaves are not keeled
Flowers	Petaloid staminoids are with a notched apex	Petaloid staminoids are with a rounded apex
	Flowers appear from the second corpuscle	Flowers appear (seemingly) from the first corpuscle
Fruits	Capsule outline appears hexagonal (viewed from the top)	Capsule outline appears nearly circular (viewed from the top)
	Capsules are wider than high	Capsules are higher than wide
Seeds	Seeds are light brown and smooth	Seeds are black and papillose

### Study area

The Knersvlakte is a gently undulated plain in the western part of southern Africa (30°27ʹ–32°05ʹS, 17°46ʹ–19°06ʹE). The mean annual precipitation is ~116 mm. The lowest temperatures in winter are 5–10 °C (with rare occurrences of frost days), and the highest daily mean temperatures in summer are 30–35 °C (Mucina *et al.*, 2006). Coastal fog and the frequent occurrence of dew also provide important additional sources of moisture for the plants (Desmet, 2007). The dominant soil types are shallow Leptosols, saline Solonchaks and the comparably less saline Cambisols (Schmiedel and Haarmeyer, 2010*a*, *b*).

The entire distribution range of *Oophytum* is limited to a roughly triangular area of 2500 km^2^. The *Oophytum*-inhabited quartz fields are characterised by saline soils. The soil pH of *O. nanum* habitats ranges between pH 6.5 and 7.0, whereas *O. oviforme* is typically found on slightly more acidic soils (pH 6.0–6.5) (Schmiedel, 2002). *Oophytum nanum* inhabits mainly gentle slopes or plains with a cover of smaller quartz pebbles, whereas the western populations of *O. oviforme* are often found on steep upper slopes and edges of plateaux with a higher cover of large stones (Ihlenfeldt, 1978). These western populations are geographically closer to the coast than the other populations, thus receiving slightly higher moisture levels as a result of the regular coastal fog (Ihlenfeldt, 1978).

### Sampling

Leaf samples of 35 *Oophytum* populations (ten individuals per population) were collected across the entire distribution area of the genus in the Knersvlakte, Western Cape, South Africa (CapeNature permit number AAA005-00186) (Supplementary Data Table S1). At 29 of the 35 population sampling sites, we also collected composite soil samples from 0–5 cm soil depth accompanied by a standardised set of habitat and population variables (Table 2).

**Table 2.** Nine environmental and three population-specific variables and their categorisation into classes.

	Trait name	Class 0	Class 1	Class 2	Class 3	Class 4	Class 5	Class 6	Class 7
A	pH	–	4.00–4.50	4.51–5.00	5.01–5.50	5.51–6.00	6.01–6.50	6.51–7.00	7.01–7.50
B	Electrical conductivity (mS cm^−1^)	–	1.00–5.00	5.01–10.00	10.01–15.00	15.01–20.00	–	–	–
C	Orientation to the cardinal direction	0 = no orientation	1 = south	2 = south-west	3 = west	4 = north-west	5 = north-east	6 = north	–
D	Inclination (steepness of slope) (°)	0	1–10	11–20	21–30	31–40	41–50	–	–
E	Total stone coverage (%)	–	50–60	61–70	71–80	81–90	91–100	–	–
F	Small stones (0–2 cm) (%)	(0)	1–10	11–20	21–30	31–40	41–50	51–60	>60
G	Medium stones (2–6 cm) (%)	(0)	1–10	11–20	21–30	31–40	41–50	51–60	–
H	Large stones (>6 cm) (%)	0	0.1–10	11–20	21–30	31–40	–	–	–
I	Population density (50 cm × 50 cm) (average of 3)	–	0.1–10	11–20	21–30	31–40	41–50	–	–
J	Number of leaf pairs per plant (average of ten)	–	1–5	6–10	11–15	16–20	–	–	–
K	Population area (m^2^)	–	1–200	201–500	501–1000	1001–2000	>2001	–	–
L	Position on slope	0 = flat plain	1 = foot slope	2 = mid slope	3 = upper slope	4 = hill top	–	–	–

Different numbers of samples were used for the individual methods. All populations were assigned to four geographical regions according to their location and based on first phylogenetic analyses (blue = northern group, green = central group, orange = eastern group and red = western group; Fig. 2). The samples for the outgroup taxa in the phylogenetic analyses were collected from the scientific living plant collection at the Loki Schmidt Botanical Garden of the University of Hamburg (for voucher information and GenBank accessions, see Supplementary Data Table S1).

### Sample processing

The DNA was extracted according to a modified cetyltrimethylammonium bromide (CTAB) protocol (Elling *et al.*, 2010). For the phylogenetic analyses, four chloroplast (cp) regions were chosen: partial sequences of the coding region of *mat*K, and the intergenic spacers *trn*Q-*rps*16, *trn*L-*trn*F and *trn*S-*trn*G. The PCR reagents, conditions and primers are summarised in Supplementary Data Tables S2–S4. All PCR products were purified, and both the forward and reverse amplicons were sequenced. Additional internal sequencing primers were developed for the *trn*G-*trn*S region: trnSG-514Faiz (5ʹ-CTGGCGAGGTACTGATCAGG-3ʹ) and trnSG-552Raiz (5ʹ-TATTATTCCCACGGCCTGGC-3ʹ). The sequence products were precipitated and scored on a 3500 Genetic Analyzer (Thermo Fisher Scientific Inc.) according to the manufacturer’s instructions. In addition to our 28 *Oophytum* samples collected for the present study, the published NCBI sequences of *Oophytum nanum* (‘Klak791’), *Dicrocaulon brevifolium* N.E.Br., *Diplosoma retroversum* (Kensit) Schwantes and *Monilaria moniliformis* (Thunb.) Schwantes of the ‘*Dicrocaulon* clade’ (Klak *et al.*, 2013) were included in the phylogenetic analyses. As an outgroup, we chose *Gibbaeum geminum* N.E.Br., *Malephora crassa* (L.Bolus) H.Jacobsen & Schwantes and *Delosperma spec.* (Supplementary Data Table S1).

The AFLP analyses of the whole-plant genomes according to Vos *et al.* (1995) were performed according to a protocol by Bertram (2007) modified for screening on the 3500 Genetic Analyzer. Sequences of the adapters, pre-amplification and selective-amplification primers are listed in Supplementary Data Table S2. The Eco-A+TG primer was fluorescently labelled for detection. Diluted products of selective amplification were detected on the 3500 Genetic Analyzer as described in the user manual. GeneScan™ 600 LIZ® Size Standard v.2.0 was chosen as internal size standard.

All 29 soil samples (Supplementary Data Table S1) were sieved to <2 mm grain size. For the electrical conductivity measurements, 10 g of sieved soil was mixed with 25 mL of sterile water. For the pH measurements, 10 g of sieved soil material was mixed with 25 mL of 0.01 M CaCl_2_ solution. Both solutions were shaken for 1 h before being measured with a SevenGo Duo™ pH/conductivity meter SG23 (Mettler Toledo).

### Phylogenetic analyses

Each forward and reverse sequence was individually edited and a consensus sequence per chloroplast-marker and sample was generated with Sequencher v.5.4.6 (Gene Codes Corporation, Ann Arbor, MI, USA). Consensus sequences of each chloroplast region were aligned with MAFFT v.7.475 (Katoh *et al.*, 2019) and manually corrected in MEGA v.11 (Tamura *et al.*, 2021). All four alignments were concatenated to a single nexus file using SequenceMatrix (Vaidya *et al.*, 2011). A TCS haplotype network analysis (Clement *et al.*, 2000) was conducted with PopArt (Leigh and Bryant, 2015). An unrooted phylogenetic network was calculated with Splitstree v.5 (Huson and Bryant, 2006).

For all four alignments, we performed a gap coding with FastGap v.1.2 (Borchsenius, 2012). The merged chloroplast regions and the gap-coded partition matrix were taken for the Bayesian inference (BI) analysis conducted with MrBayes v.3.2.7 (Ronquist *et al.*, 2012). A Markov chain Monte Carlo (MCMC) run with four chains was carried out for 5 million generations. The nst-mixed model of evolution was chosen for the DNA partitions, whereas the variable model was chosen for the gap partitions. In both instances, the gamma rates were chosen. One million trees were saved per run. The resulting topologies were analysed with the MrBayes sump and sumt commands and with a relative burn-in of discarding the first 25 %. The resulting 50 % majority consensus tree was visualised with Figtree v.1.4.4 (Rambaut, 2018) and modified with Affinity Photo v.1.10.6.1665. In addition, the tree was processed with Mesquite v.3.70 (Maddison and Maddison, 2021) and the Package Cartographer v.1.52 (Maddison and Maddison, 2017) to project the BI tree onto a map. Maximum parsimony (MP) analyses were performed with PAUP v.4.0a169 (Swafford, 2002), with the following settings: hsearch start = stepwise, addseq = random, nreps = 1000, swap = TBR, multrees = yes and for bootstrap nreps = 10000, grpfreq = no search = heuristic/addseq = random, nreps = 1000, swap = tbr, rearrlimit = 1000, hold = 1. For the maximum likelihood (ML) analyses, we chose raxmlGUI v.2.0 (Edler *et al.*, 2020), which included Modeltest-NG (Darriba *et al.*, 2020) and RAxML-NG v.1.1.0 (Kozlov *et al.*, 2019). For the ML analyses, we chose ML + transfer bootstrap expectation + consensus and a bootstrap of 10 × 1000 run mode. *Delosperma spec.* has been selected as the outgroup in all phylogenetic analyses.

### AFLP analyses

AFLP products were detected with GeneMapper® v.4.1 (Thermo Fisher Scientific Inc., Waltham, MA, USA). All samples were checked at least twice, and only reproducible PCR products were included in a 1/0 matrix in MS Excel 2016. Reproducible peaks were coded as ‘1’ and peak absence as ‘0’. We performed statistical analyses of the overall population dataset and the four geographical clusters separately in AFLPsurv v.1.0 (Vekemans, 2002). To estimate the relationship of linear genetic to geographical distances, a Mantel test (Mantel, 1967) was performed with GenAlEx v.6.51b2 (Peakall and Smouse, 2012). The results of the Mantel test were visualized in a scatter diagram. An analysis of molecular variance (AMOVA; Excoffier *et al.*, 1992) was calculated with GenAlEx v.6.51b2 (Peakall and Smouse, 2012) to identify the genetic exchange rate among the four geographical regions, among and within populations. In addition, we calculated the band frequency, the allele frequencies, the diversity and the Shannon index (Sherwin *et al.*, 2006) by applying GenAlEx v.6.51b2 (Peakall and Smouse, 2012). We conducted a principal coordinate analysis (PCoA) with the algorithm of Dice (1945) in PAST v.4.11 (Hammer *et al.*, 2001). The populations were grouped according to their geographical regions (north, centre, east and west). Genetic grouping was visualised in a network analysis of Splitstree v.5 (Huson and Bryant, 2006). Furthermore, we determined the rate of genetic exchange between populations and a one-way PERMANOVA (Anderson, 2001) to assess the significance of population differentiation with PAST v.4.11 (Hammer *et al.*, 2001).

With the AFLP dataset, a BI analysis was conducted with the restriction site model and the option of noabsencesites of the MrBayes v.3.2.7 software (Ronquist *et al.*, 2012). The Dirichlet prior for the state frequencies was set to (3.00, 1.00), matching the actual 0/1 frequencies in our dataset. Given that the AFLP dataset did not contain an outgroup, an unrooted tree was selected for the evaluation of the majority rule consensus tree with FigTree v.1.4.4 (Rambaut, 2018).

A Bayesian clustering approach was performed with the admixture model and the LOCPRIOR parameter (Hubisz *et al.*, 2009) for the analyses with STRUCTURE v.2.3.4 (Falush *et al.*, 2007). For the Markov chain Monte Carlo run, the burn-in 100 000 repeats and *K*1–*K*19 with 20 iterations were chosen (K = true number of clusters). The admixture model and *K*1 to one *K* higher than the number of respective populations were selected for the analyses of the populations in each region. The results were summarised with STRUCTURE HARVESTER (Earl and von Holdt, 2011). The respective files for the highest Δ*K* values were summarised further with CLUMPP (Jakobsson and Rosenberg, 2007) and visualised with DISTRUCT v.1.1 (Rosenberg, 2004). In addition to barplots, pie charts were generated for the highest Δ*K* value of the overall analysis from the resulting CLUMPP popfile in Excel 365 and Affinity Photo, which were displayed on a map created with QGIS v.3.22.

### Environmental variables and principal component analysis

In Table 2, nine environmental variables and three population-specific variables were summarised from the 29 study sites. The nine environmental variables were soil pH, soil electrical conductivity, orientation to the cardinal point, slope (steepness of the slope), position on the slope, total stone cover, and cover of the three stone size classes 0–2, 2–6 and >6 cm. The three population-specific variables were population density (average), leaf pairs per plant (average) and population area. Each of the variables (Table 2) was coded into classes to facilitate the analyses for the principal component analysis (PCA) and the comparative heatmap analyses. Based on these 12 variables per study site, we performed a PCA with PAST v.4.11 (Hammer *et al.*, 2001) with the settings: Matrix = variance-covariance, Groups = disregard, Missing values = iterative imputation, Bootstrap = 10 000.

### Comparative analyses of AFLP data, four chloroplast DNA markers and 12 environmental and population-specific variables

We visualised the genetic data in combination with the nine environmental and three population-specific variables per population to identify groupings of *Oophytum* populations according to their genetic background and habitat conditions using heatmaps. For this purpose, we carried out two different heatmap analyses with the web tool ClustVis software (Metsalu and Vilo, 2015). Specifically, both datasets [AFLP and chloroplast DNA (cpDNA)] were calculated independently with the following clustering methods: Clustering distance for rows and columns = maximum, Clustering method for rows and columns = complete, and tree ordering options for rows and columns = tightest cluster first. For the heatmap calculation of the combined AFLP and ecological data, the CLUMPP-pop file for *K* = 3 (based on the STRUCTURE HARVESTER result) was selected. For the calculations of the cpDNA heatmap, a general time-reversible distance matrix was calculated in PAUP v.4.0a169 (Swafford, 2002) with default settings and incorporated into the heatmap analysis.

## RESULTS

### *Phylogenetic analyses of the genus* Oophytum

**Fig. 3.**
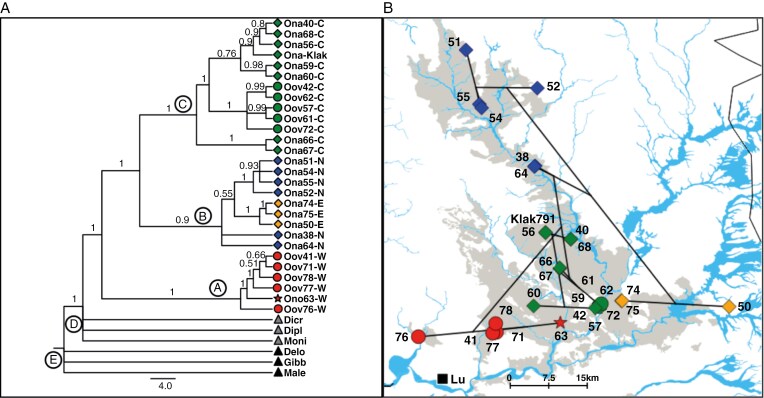
(A) Bayesian inference 50 % majority-rule consensus tree of 28 *Oophytum* samples (clades A–C), three members of the *Dicrocaulon* clade (clade D) and three outgroup samples (clade E), using a combined alignment of four plastid regions (*mat*K, *trn*L-*trn*F, *trn*Q-*rps*16 and *trn*S-*trn*G). Numbers above lines indicate the posterior probability values of 5 Million replicates, Ona = *Oophytum nanum*, Ono = ‘*O. nordenstamii*’, Oov = *O. oviforme*, Moni = *Monilaria moniliformis*, Dicr = *Dicrocaulon brevifolium*, Dipl = *Diplosoma retroversum*, Gibb = *Gibbaeum geminium*, Male = *Malephora crassa*, Delo = *Delosperma spec*. (B) Section of the Bayesian inference consensus tree with only the *Oophytum* clades (A–C) plotted on the distribution map of *Oophytum* (Fig. 1B) conducted with Mesquite Cartographer and QGIS v.3.22.0 (blue diamonds = northern group of *O. nanum*, green diamonds = central group of *O. nanum*, orange diamonds = eastern group of *O. nanum*, green circles = central group of *O. oviforme*, red circles = western group of *O. oviforme*, red star = ‘*O. nordenstamii*’ population, black square = village, Lu = Lutzville. Number beside symbol is the population number).

Sequencing of the four plastid markers of 28 *Oophytum* samples and six outgroup samples resulted in an alignment of 3498 bp in total, of which 100 positions were parsimony informative (37 substitutions and 63 gaps; Table 3). The genus *Oophytum* was monophyletic in all phylogenetic analyses. For the BI tree, the support value for the monophyly of the genus was strong [posterior probability (PP) = 1], although the placement of *Oophytum* within the *Dicrocaulon* clade, which consisted of the genera *Dicrocaulon*, *Diplosoma*, *Monilaria* and *Oophytum*, remained unresolved (Fig. 3A). This was also confirmed by the MP and ML analyses (Supplementary Data Fig. S1).

**Table 3.** Information about the four plastid regions, the alignments and their gaps.

	*mat*K	*trn*L-*trn*F	*trn*Q-*rps*16	*trn*S-*trn*G	Total
Total length of alignment (bp)	773	941	925	857	3496
Parsimony-informative sites (bp)	3	3	20	11	37
Singleton (bp)	12	22	37	29	100
Variable sites (bp)	15	25	57	43	138
Conserved sites (bp)	758	902	835	781	3276
Total number of gaps	2	37	49	32	120
Number of informative gaps	2	20	25	16	63

In all phylogenetic analyses, the genus was divided into three major clades by two nodes, which did not reflect the two morphologically defined species: clade A was clearly separated from the rest of the *Oophytum* samples (PP = 1), which were further split into the sister clades B and C (PP = 1). These three clades reflected regional groups (Fig. 3B). Clade A (western clade) was placed at the base of the genus, comprising *O. oviforme* samples from the western distribution area and the ‘*O. nordenstamii*’ population. The basal position within clade A was occupied by the westernmost sample (*O. oviforme* 76) followed by the ‘*O. nordenstamii*’ 63 (red star in Fig. 3) as sister to the remaining samples of the western clade. Clade B (northern and eastern clade) included samples from all northern and eastern *O. nanum* populations with a moderate support of PP = 0.9. However, the grouping of the eastern samples within the northern samples of this clade was not supported (PP = 0.55). The position of the two samples 38 and 64, which connect the northern and central populations geographically, remained unresolved within this clade. The well-supported clade C (central clade, PP = 1) comprised all populations of *O. nanum* and *O. oviforme* from the centre. Clade C was further divided into three subclades, with very good (PP = 1) to poor support (PP = 0.76). We conducted further phylogenetic analyses (MP and ML, with and without FastGap coding and with and without poly positions) (Supplementary Data Fig. S1). The ML and MP phylogenetic trees also resolved *Oophytum* into the three clades, but only the FastGap-coded trees of the BI and MP analyses revealed well-supported grouping within the subclades, especially in clade C.

**Fig. 4.**
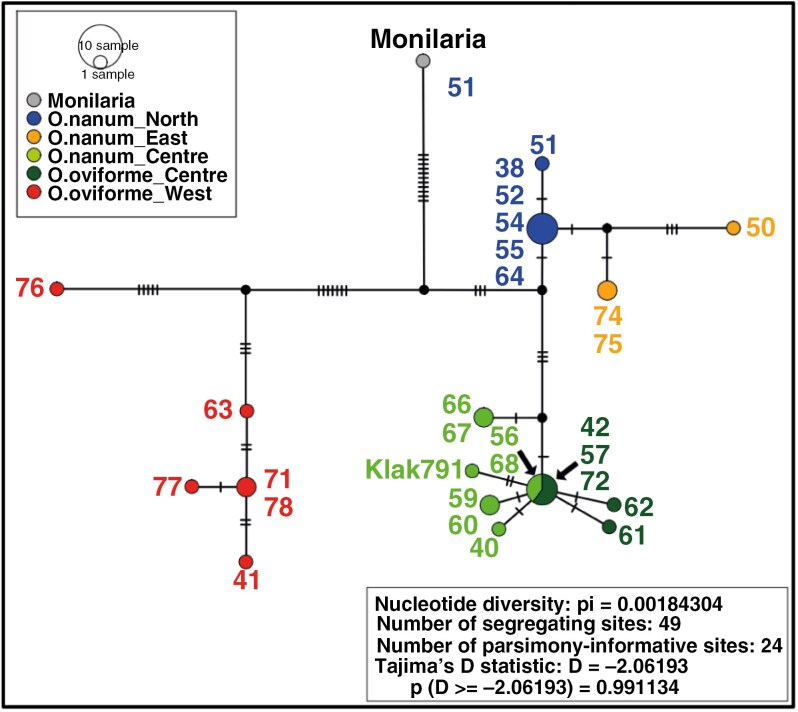
TCS haplotype networks. Network of 28 *Oophytum* samples and one *Monilaria moniliformis* sample. Black dots represent an unknown haplotype created by PopArt. Mutation steps are indicated by a black line between the haplotypes. The respective population numbers are next to the haplotype. The statistics values can be seen in the lower right box.

The TCS haplotype network based on 24 parsimony-informative sites revealed 22 chloroplast haplotypes, of which five were unknown or probably extinct ancestral inferred haplotypes (Fig. 4). All detected *Oophytum* haplotypes showed a geographical clustering within the study area that also did not reflect the two morphological species of the genus. The western samples were separated from all other *Oophytum* samples by three of the five probably extinct ancestral haplotypes. Additionally, the outgroup sample *Monilaria moniliformis* was connected to the middle of these missing haplotypes. A similar picture was apparent in the unrooted Splitstree v.5 network (Supplementary Data Fig. S2), where the outgroup was positioned between the two lineages of *Oophytum*. The western *Oophytum* samples (Fig. 4, marked in red) formed a separate evolutionary lineage, whereas the single ‘*O. nordenstamii*’ (63) sample and the four *O. oviforme* samples were separated from the westernmost *O. oviforme* population (76) via an unknown haplotype and eight mutation steps.

The second evolutionary lineage (Fig. 4, right side) split into two branches that on one side represented the northern and eastern group (blue and orange) and on the other side the central group (green). The central group was divided into a haplotype shared by two samples (66 and 67) and a major haplotype via another unknown ancestor. The major haplotype was shared by five samples of *O. nanum* (56 and 68 = bright green) and *O. oviforme* (42, 57 and 72 = dark green). Four private haplotypes (Klak791, 40, 61 and 62) and one shared haplotype (from samples 59 and 60) were also connected directly to this major haplotype. The northern group (marked in blue) possessed one main haplotype, from which the northernmost sample 51 and the three eastern samples (marked in orange) diverged. The neighbouring eastern samples (74 and 75) and the easternmost sample (50) were separated from the northern major haplotype via another unknown ancestral haplotype. The statistical value of Tajima’s *D* was highly negative (−2.06), which could indicate a bottleneck effect but was not significant (*P* = 0.99).

### *Population genetics of the genus* Oophytum

#### Population structure.

**Fig. 5.**
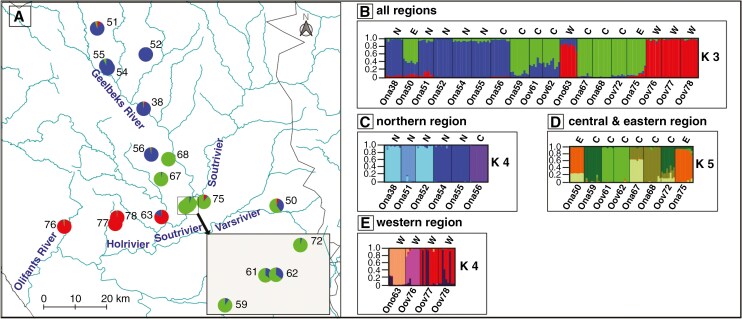
STRUCTURE (CLUMPP-DISTRUCT) results of the AFLP dataset from all 18 *Oophytum* populations in the whole study area. (A) Structure barplot for the highest Δ*K* value (*K* = 3) were translated into pie charts and plotted on a geographical map. (B) Barplot for best Δ*K* = 3 of all 18 populations. (C) Barplot for best Δ*K* = 4 of six northern populations region. (D) Barplot for best Δ*K* = 5 of six central and two eastern populations. (E) Barplot for best Δ*K* = 4 of four western populations.

The 1/0 AFLP matrix of 163 individuals representing 18 populations resulted in 264 reproducible loci. The STRUCTURE analysis of all 18 populations resulted in a best Δ*K* value of three (Supplementary Data Fig. S3), which corresponded to the tripartite phylogenetic trees and cp haplotype analysis (Figs 3 and 4). This partition also did not correspond to the taxonomical species concept of *Oophytum* (Fig. 5A, B; Supplementary Data Fig. S4). Eleven of 18 populations had pure to almost pure gene pools, four populations (59, 61, 62 and 63) had a mixture of two gene pools, and three populations [the northernmost (51) and the two eastern populations (50 and 75)] had three gene pools. Among the populations that possessed two or three gene pools, one gene pool always dominated, and the others were present in only very small proportions. The gene pool marked in red represented all three western *O. oviforme* populations (76, 77 and 78), which were almost pure. Also, the ‘*O. nordenstamii*’ population (63) had this western gene pool but admixed with the gene pool marked in blue. This blue gene pool was present in nearly all *O. nanum* populations, but dominant in all northern populations, either as a pure gene pool (52) or with very low introgression of other gene pools. Population 56 is assigned to this northern gene pool in this analysis. This is in conflict with the phylogenetic analyses (Fig. 3A), in which this population clustered in the central clade C. Geographically, population 56 links the northern and central populations. The second discrepancy is the position of the eastern populations (50 and 75). In contrast to the phylogenetic analysis where populations 50 and 75 were placed into clade B (Fig. 3A), the STRUCTURE analysis revealed their integration into the central populations (Fig. 5). In accordance with the phylogenetic analyses, the three central *O. oviforme* populations (61, 62 and 72) here also belong to the central (eastern) *O. nanum* gene pool, which is genetically distinct from the western *O. oviforme*.

Each of the three regional gene pools identified in the overall STRUCTURE analysis was analysed individually (Fig. 5C–E; Supplementary Data Figs S5–S8). For the six northern populations, a best Δ*K* of four gene pools was identified (Fig. 5C), with shared gene pools between the populations 54 and 55 and in populations 38 and 52. The remaining two populations (51 and 56) each had their own private gene pool. All four gene pools were almost pure, with minimal influences of other gene pools. In the central and eastern region, the eight populations comprised five different gene pools (best Δ*K* = 5; Fig. 5D), which were more admixed. Three populations were hybrids with a stable proportion of different gene pools. Two populations (50 and 67) were hybrids of two gene pools, and population 72 had contributions from three different gene pools. These three populations were mixed with the main gene pool of population 67 (light olive green), and two of these hybrids (67 and 72) were further mixed with the gene pool of population 68 (olive green). The eastern populations shared one main gene pool (orange), also to varying proportions. Likewise, the two neighbouring *O. oviforme* populations 61 and 62 shared one gene pool (bright green), which was almost pure. Populations of the western region revealed a best *K* = 4 (Figs 5E; Supplementary Data Fig. S8). The neighbouring populations 77 and 78 shared a common gene pool, whereas the westernmost (76) and the ‘*O. nordenstamii*’ population (63) each had a private gene pool. The fourth gene pool was interspersed in all four populations, but within single individuals and in considerably varying quantities.

**Fig. 6.**
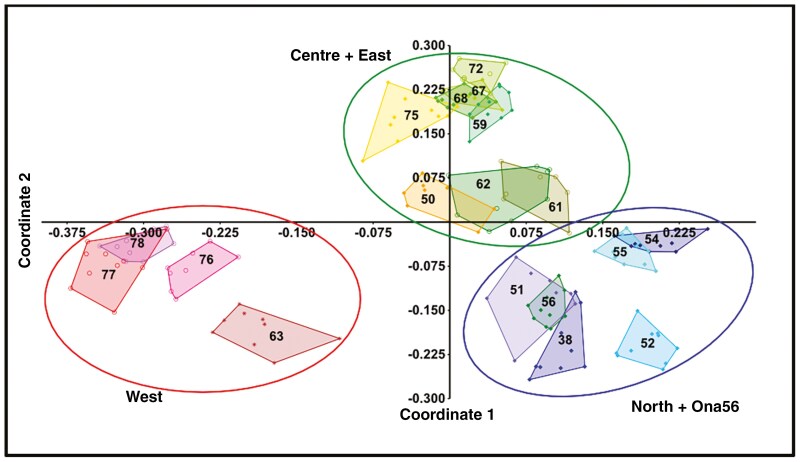
Principal coordinate analysis (PCoA) of the AFLP dataset from all 18 *Oophytum* populations conducted with PAST. Northern populations are coloured in blue, central populations in green, eastern populations in orange and western populations in red. Numbers inside the polygons are the population identities.

The PCoA, based on AFLP data, again revealed a geographical tripartite division of the 18 *Oophytum* populations instead of following the taxonomic two-species concept (Fig. 6). Within each of the three regional main groups, two to four separate subgroups were identified. The western group (encircled in red) was clearly separated from the other two groups (northern and centre and eastern). From these four western populations, only populations 77 and 78 overlapped slightly, while populations 76 and 63 were distinctly separated. The central and eastern group (encircled in green) was divided into two subgroups, whereby the two eastern populations were each assigned to one group. The northern group (encircled in blue) comprised three distinct units: population 52 was separate, the neighbouring populations 54 and 55 marginally overlapped, and all of them were separate from the remaining northern populations.

The population structure of the 18 *Oophytum* populations revealed similar genetic patterns to those shown by the BI and the Splitstree v.5 network analyses (Supplementary Data Figs S9 and S10).

#### Genetic exchange and diversity analyses.

According to the AMOVA of 18 populations, the genetic exchange within populations (41 %) and among populations (42 %) was significantly higher than the exchange among regions (17 %) (Table 4; Supplementary Data Fig. S11). Wright’s fixation index (*F*_ST_) also provided information of the gene flow between populations, where an index ranging from zero for panmixia to one for complete genetic separation of populations is assumed. In our analysis, the regional *F*_ST_ values ranged from 0.3159 (northern populations) to 0.4013 (central populations) (Supplementary Data Table S5). The overall analysis revealed the highest *F*_ST_ value of 0.4225 for all 18 populations. These data indicate a significantly strong differentiation of populations within *Oophytum*. The genetic variance estimated by PhiPT (genetic variance within populations) values (PhiPT is an analogue of Wright's fixation index and also estimates genetic differention among populations) was almost constant within the three peripheral regions, ranging from 0.457 in the eastern population to 0.479 in the western population, but was highest in the centre (0.555). The overall analysis of all 18 populations across the entire study area also revealed a highly differentiated genetic structure of *Oophytum* (PhiPT = 0.585; Table 4).

**Table 4.** Analysis of molecular variance (AMOVA) of 18 *Oophytum* populations together and for all populations of each region individually, indicating variance among four geographical regions, among and within populations; d.f. = degrees of freedom, SS = sum of squared observations, MS = mean sum of squared observations, Est. Var. = estimated variance, % = percentage variance, PhiRT = genetic variance among regions, PhiPR = genetic variance among populations, PhiPT = genetic variance within populations. All values of Phi were significant (*P < 0.01).

	d.f.	SS	MS	Est. Var.	Percentage	PhiRT	PhiPR	PhiPT
All 18 populations								
Among regions	3	1122.696	374.232	5.888	17	–	–	–
Among populations	14	2027.335	144.810	14.447	42	–	–	–
Within populations	145	2088.967	14.407	14.407	41	–	–	–
Total	162	5238.998	–	34.742	100	0.169*	0.501*	0.585*
Five northern populations								
Among populations	4	598.809	149.702	14.184	46	–	–	–
Within populations	42	697.910	16.617	16.617	54	–	–	–
Total	46	1296.718	–	30.801	100	–	–	0.461*
Seven central populations								
Among populations	6	930.786	155.131	16.102	56	–	–	–
Within populations	55	709.170	12.894	12.894	44	–	–	–
Total	61	163.956	–	28.996	100	–	–	0.555*
Two eastern populations								
Among populations	1	147.190	147.190	14.609	46	–	–	–
Within populations	16	277.260	17.329	17.329	54	–	–	–
Total	17	424.450	–	31.938	100	–	–	0.457*
Four western populations								
Among populations	3	350.550	116.850	11.602	48	–	–	–
Within populations	32	404.628	12.645	12.645	52	–	–	–
Total	35	755.177	–	24.247	100	–	–	0.479*

Additionally, the Mantel test revealed an interrupted gene flow between populations (Supplementary Data Fig. S12). All samples were clearly arranged in vertical rows. Thus, the geographical distance corresponded to the genetic distance between each population. Furthermore, the one-way PERMANOVA with Bonferroni corrections exhibited a significant limited gene flow (*P*-values of <0.05) between all *Oophytum* populations except between Ona51 and Oov72 (*P* = 0.1377), Ona50 and Ona51, Oov61 and Oov72 and between Ono63 and Oov78 (*P* = 0.0612 each) (Supplementary Data Table S6).

Further analyses of the AFLP data revealed very low genetic diversity within all 18 *Oophytum* populations (Supplementary Data Tables S7 and S8). The mean diversity (*h*) and unbiased diversity value (*uh*) were below 0.100 and 0.110, ranging between 0.069/0.080 in population 72 and 0.163/0.189 in population 51, respectively (Supplementary Data Table S7). In addition, populations 38, 75 and 50 (easternmost population) exhibited slightly higher genetic diversity rates.

Including the evenness and allele frequencies in the genetic diversity calculation of each population revealed a mean Shannon’s information index (*I*) of 0.148, with the highest genetic diversity within the northernmost population 51 (*I* = 0.247), again followed by population 38 in the north (*I* = 0.163) and populations 50 and 75 from the east (0.170–0.182) (Supplementary Data Table S8). Likewise, population 72 had the lowest diversity rate (*I* = 0.107).

### *The environmental and population-specific variables of* Oophytum

**Fig. 7.**
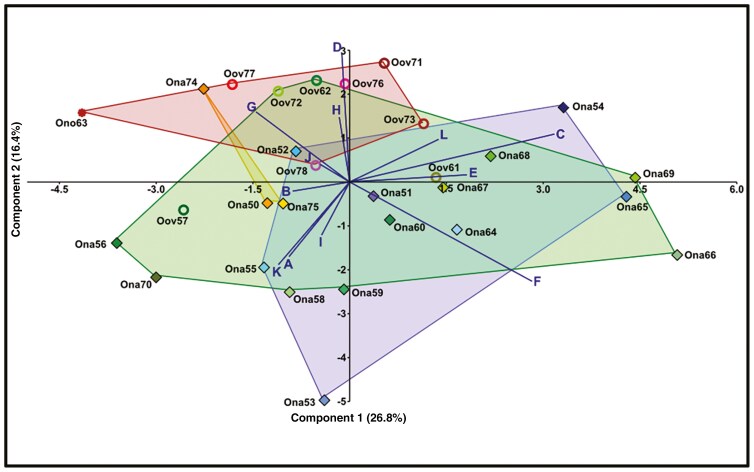
Principal component analysis (PCA) of nine environmental variables (six soil and three topographical variables) and three population-specific variables from 29 *Oophytum* populations. The samples were grouped according to four geographical regions. Ona = *O. nanum*, Ono = ‘*O. nordenstamii*’, Oov = *O. oviforme*. Numbers beside coloured symbols are the population numbers. Blue diamonds = northern *O. nanum* populations, green diamonds = central *O. nanum* populations, green circles = central *O. oviforme* populations, orange diamonds = eastern *O. nanum* populations, red circles = western *O. oviforme* populations, red star = ‘*O. nordenstamii*’ population. Biplots: A = pH value, B = electrical conductivity EC (in millisiemens per centimetre), C = orientation to the cardinal direction, D = inclination (steepness of slope, in degrees), E = total stone coverage (as a percentage), F = 0–2 cm stones (as a percentage), G = 2–6 cm stones (as a percentage), H = >6 cm stones (as a percentage), I = population density (average), J = leaf pairs per plant (average), K = population area (in metres squared), L = position on slope.

The PCA based on nine environmental and three population-specific variables of 29 *Oophytum* populations showed the western *O. oviforme* populations to be more strongly associated with steepness of slope (biplot D) and soil cover by large stones (biplots G and H) (Fig. 7). Otherwise, the populations did not show a strong association to the other habitat conditions. The plants in these populations also differ slightly in the average of leaf pairs per plant (biplot J). The central and northern populations, in turn, largely overlapped in the ordination space and did not show an association with any of the environmental variables (Fig. 7). The variables describing the population extent (biplot K), soil pH (biplot A) and density (biplot I) only had low explanatory values (Fig. 7). The two axes of the ordination, however, explained only a very small amount of the total variance in the dataset (axis 1 = 26.8 % and axis 2 = 16.4 %).

### Comparative analyses of environmental and population-specific variables and genetics

**Fig. 8.**
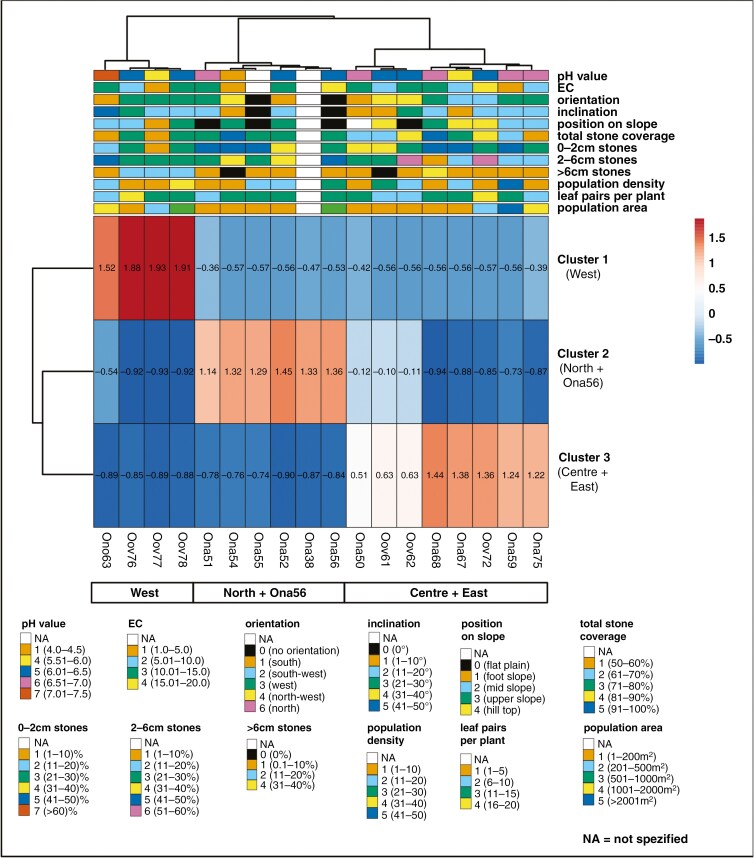
ClustVis heatmap of a comparative analysis with the AFLP-CLUMPP-pop.file for *K* = 3 and 12 habitat and population-specific variables for 18 *Oophytum* populations. Ona = *O. nanum*, Ono = ‘*O. nordenstamii*’ and Oov = *O. oviforme*.

The comparative heatmap analysis of AFLP data with the nine environmental and three population-specific variables revealed three major clusters based on AFLP data genetic distances (Fig. 8). These clusters were separated into a western *O. oviforme* cluster (cluster 1), a northern *O. nanum* cluster (cluster 2) and a central and eastern cluster consisting of *O. nanum* and *O. oviforme* (cluster 3). In cluster 1, the western populations with apparently high positive values (Fig. 8, indicated in dark red) differed from the other two groups, which ranged more towards the lower end of the scale (blueish colour). Only ‘*O. nordenstamii*’ population 63 segregated marginally (lighter reddish colour). Within the central and eastern clade of cluster 3, the populations 50 (*O. nanum*) and 61 and 62 (*O. oviforme*) showed relatively low positive values, indicative of a slight differentiation. None of the environmental variables could explain any of the three genetic groups. Only the habitat of ‘*O. nordenstamii*’ (63) differed from the other populations by a higher soil pH and steepest inclination. All others comprised a mosaic of different habitat conditions on each quartz field.

**Fig. 9.**
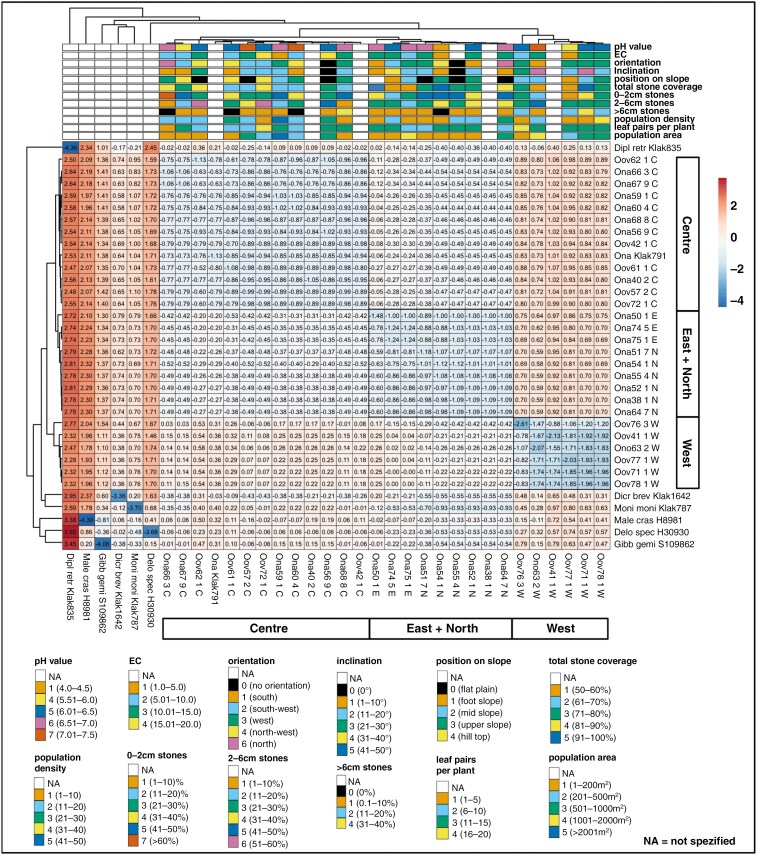
ClustVis heatmap of a comparative analysis with a four chloroplast-marker genetic GTR- distance (GTR = general-time-reversible model of DNA sequence evolution) including 34 samples (28 *Oophytum* samples, 3 *Dicrocaulon* clade samples and 3 outgroup samples) and 12 habitat and population-specific variables. Ona = *O. nanum*, Ono = ‘*O. nordenstamii*’ and Oov = *O. oviforme*. Black frames in the heatmap have been added subsequently for better recognisability.

The heatmap of the environmental and population-specific variables in relationship to the phylogenetic distances of the cpDNA data revealed a comparable grouping of the *Oophytum* samples with the centre, east and north, and west clusters (Fig. 9). The outgroups were separated, and the distinction between the three *Oophytum* groups was clearly visible by a diverging colouring. The western populations also differed clearly from the other two groups, recognisable by slightly darker blue tones in the lower right part or the slightly darker reddish colour in the two groups above. Additionally, a clear trend in the grouping of the environmental and population-related variables in combination with cpDNA data could not be identified.

## DISCUSSION

### Genetic structure vs. current taxonomy of the genus *Oophytum:* speciation of *O. oviforme*

The overarching outcome of all our analyses was a basal division of the genus *Oophytum* into three major genetic groups. These three groups clustered according to geographical patterns, which do not match the classification into the two taxonomically described *O. nanum* and *O. oviforme* species of the genus. This separation was visible in the two independent cpDNA and whole-genome AFLP analyses. The split of the western *O. oviforme* from all other *Oophytum* taxa is supported by a private gene pool of western *O. oviforme*.

This speciation of the western *O. oviforme* within the genus is characterised by the basal position of clade A in the phylogenetic trees and a separated evolutionary line in the haplotype analysis. The western *O. oviforme* split off from a first unknown, putatively extinct common *Oophytum* ancestor that diverged from the outgroup (*Monilaria moniliformis*). Two further ancestor haplotypes separated the western *O. oviforme* from the remaining members of the genus, indicating a very early speciation of the two species in the evolution of *Oophytum*.

The western region is characterised by comparatively higher humidity due to coastal fog (Desmet, 2007). The respective *Oophytum* habitats are situated on steeper quartz fields with larger stones (Fig. 7), which provide more run-off of morning dew that moistens the soil and can serve as a soil water resource for the shallow-rooting dwarf succulents (Musil *et al.*, 2005; Eibes *et al.*, 2022). This supports the hypothesis that the genetic isolation might be linked to an adaption to slightly more humidity (Figs 8 and 9).

The evolution of all western *O. oviforme* appears to have occurred via ‘*O. nordenstamii*’ (population 63), which is geographically located between the *O. oviforme* and *O. nanum* distribution areas (Fig. 5). It was accompanied by the loss of the putatively primary/ancestral *Oophytum* gene pool characterising the northern *O. nanum* populations (17 %; Fig. 5). This loss might have been caused by a bottleneck in the founder population of *O. oviforme* and might be the price of adaptation to the more beneficial habitat in the western region.

It is also possible that the ‘*O. nordenstamii*’ population might be a relic hybrid of *O. oviforme* and *O. nanum*. This population grows on a quartz field with neutral soil pH (Figs 8 and 9) and might benefit from easier uptake of nutrition from the less acidic soil. Whether or not the white-flowering ‘*O. nordenstamii*’ could be considered as a ‘cryptic species’, according to Larsen (1957) and reviewed by Bickford *et al.* (2007), remains unresolved and requires further studies, e.g. comparative interbreeding tests with both species of *Oophytum*.

### *Genetic fragmentation of the genus* Oophytum*: a split within* O. nanum

The remaining *Oophytum* populations were separated into a northern clade and a central clade in all genetic analyses with a high support. However, there were two discrepancies between the cpDNA and the whole-genome AFLP results. In the cpDNA analyses, the eastern samples (50, 74 and 75) lay within the northern clade B, although without support (Fig. 3) and with a further deviation in the haplotype analysis (Fig. 4). In the whole-genome analyses, the eastern populations were integrated into the central group (Fig. 5A, B).

This discrepancy might be caused by an introgression of parts of the chloroplast genome from the northern into the central gene pool. Given that chloroplasts are inherited maternally, this introgression must have occurred by seed dispersal.

The second discrepancy concerned population 56, which connected the northern and central regions geographically. In the chloroplast phylogeny, this population clustered in the central clade C with moderate support (Fig. 3). In the whole-genome AFLP results (Figs 5 and 6) it was included in the northern region with an almost pure private gene pool. This indicates a further introgression of maternal cpDNA (via seeds), but in this case from the central into the northern populations. Introgressions are widespread in nature. Even before the emergence of molecular studies, several examples of introgressive hybridisations were discussed by Anderson (1949). Since then, diverse genetic studies revealed a wide variety of introgression events in very different relationships, such as in the *Heuchera* group (Soltis and Kuzoff, 1995) and in *Pilosella* hawkweeds (Fehrer *et al.*, 2007). Overall, a clear split of *O. nanum* into two main gene pools with occasional genetic exchange of cpDNA in both directions was apparent, which is presumably driven by seed dispersal. These independent introgressions of chloroplast genomes would also explain the poor support in the phylogenetic trees and the network-like branching in the Splitstree analysis within the north-eastern clade. Based on this clear separation of *O. nanum* into two genetic and geographical groups, the next step would be to provide a new species description of the northern group, while the earlier description of *O. nanum* (based on the type of R. Schlechter 8318, collected in Zoutrivier, Knersvlakte, South Africa, 28 July 1896, Isotype BOL) will remain valid for the central group. In memory of the taxonomist and initiator of this work, Professor Dr Hans-Dieter Ihlenfeldt (1932–2023), we propose ‘*Oophytum ihlenfeldtianum*’ as the name for the new species.

### *Further differentiation of* Oophytum *populations into intra-regional subgroups*

Our population genetic analyses clearly revealed a very low genetic diversity within quartz field populations. According to Ihlenfeldt (1994), recurrent droughts regularly lead to complete or nearly complete collapse of populations. Re-establishments by surviving plants or the soil seed bank decreases the genetic diversity by founder effects and genetic drift (Ihlenfeldt, 1994). Extremely low genetic diversity, accompanied by very limited gene flow, was also detected between populations of *Oophytum* (e.g. AMOVA results). The *Oophytum* metapopulation is split into single island populations with private gene pools or very small groups within all three regions, especially of neighbouring populations (Fig. 5C–E, e.g. populations 54 and 55, 61 and 62, and 77 and 78).

Small-scale genetic subdivisions in the Knersvlakte were also found in other studies of Aizoaceae, e.g. for *Ruschia burtoniae* L.Bolus and *Conophytum calculus* (A.Berger) N.E.Br. (Musker *et al.*, 2020), and for *Argyroderma* N.E.Br. (Ellis *et al.*, 2006, 2007; Boucher *et al.*, 2024). In *Argyroderma*, Ellis *et al.* (2006) also identified almost pure gene pools within restricted areas. Similar genetic patterns have also been reported for *Conophytum* N.E.Br. species on inselbergs in the Bushmanland of South Africa (Powell *et al.*, 2019) or *Lithops* N.E.Br. species in Namibia (Loots *et al.*, 2019*a*, *b*).

The splitting of the *Oophytum* metapopulation into diverse gene pools is caused by a strongly restricted gene flow due to reproductive isolation (RI) barriers. In a recent study by Boucher *et al.* (2024), an almost complete RI in species pairs of *Argyroderma* in the Knersvlakte was found, which was a result of three different pre-mating barriers: an isolation by distance (IBD) with geographical isolation on spatial scales of ~10 km, a phenological isolation by time (IBT) due to different flowering times, and an isolation by ecology (IBE) due to contrasting edaphic differences on spatial scales of only a few metres. In the study by Musker *et al.* (2020), populations of the fruticose shrub *R. burtoniae* were isolated by different habitat conditions per site (IBE), whereas for the dwarf shrub *C. calculus* IBD was more important.

Many studies about RI barriers in plants identified multiple pre- and postzygotic RI barriers, which act individually on speciation processes (Lowry *et al.*, 2008; Christie *et al.*, 2022). Often, RI barriers affect speciation individually or in combination. A very important factor in a highly heterogeneous landscape is the IBE (Wang and Bradburd, 2014), whereas IBD has an impact on island populations or island-like distributed populations (Wright, 1943). It is most likely that several of these factors together contributed to the evolution of the genus *Oophytum*.

In the western *O. oviforme* populations, two different RI barriers might play a major role. An IBD seems to be one key barrier. The distances between quartz field habitats vary between a few metres and several kilometres (Oldeland *et al*., 2022). The distance can limit both pre- and postzygotic gene flow. *Oophytum* is insect pollinated, and prezygotic genetic exchange depends on the flight distance of pollinators. *Oophytum* flowers during the winter-rainfall season and its pollinators are mainly winter-active bee species with short flight ranges. The frequent cold and wet weather conditions are unfavourable for insects and result in a short flight activity period per day (Struck, 1992; Linder *et al*., 2010). This leads to a comparatively low pollen flow among plant populations. In addition, self-sterility and ‘mass flowering’ greatly promote genetic exchange within a population rather than between populations (Ihlenfeldt, 1994). This facilitates genetic isolation in the geologically and climatically fragmented landscapes in the Cape (Linder *et al*., 2010).

Seed dispersal is a postzygotic vector, which is also strongly related to IBD. The hygrochastic capsules of *Oophytum* are optimised for seed dispersal in narrowly defined habitats within the quartz islands. This antitelechory ensures the main propagation within a proven habitat near the mother plant (Parolin, 2001). However, it considerably reduces the dispersal range, which results in limited gene flow between the island-like populations. Further secondary dissemination strategies by water run-off and dust storms offer opportunities for occasional medium- and long-distance dispersal, although the chances of reaching a suitable habitat are very low (Parolin, 2001).

The westernmost population 76, with its private gene pool, might be the product of a rare long-distance dispersal. This population is located on an isolated quartz field that is 14 km apart from the main centre of western quartz fields. A strong easterly wind (Kruger *et al.*, 2010) might have transported the seeds to this remote location and, due to its long-term isolation, the founder population might have evolved separately.

The private gene pool of the relic ‘*O. nordenstamii*’ population occurs at the eastern side of the distribution range of *O. oviforme* and could be an example of a more ecologically driven barrier. The edaphic habitat conditions were slightly different from the others. In this case, IBE seems to have played a stronger role in adaptation to these habitat conditions. Rundle and Nosil (2005) reported such cases of ecological selection resulting in barriers to gene flow.

These two isolated populations above provide a glimpse into the mechanics driving population differentiation in the western *Oophytum* populations. The metapopulation of *Oophytum* with high differentiation into 13 different gene pools (Fig. 5C–E) within such a restricted distribution area could also be the consequence of similar patterns of different RI barriers. Comparative analyses of habitat and genetic parameters revealed a heterogeneous pattern of diverse island habitats that vary slightly in their environmental conditions. Similar to the findings by Boucher *et al.* (2024), it is likely that due to a strong effect of IBD, each island population adapted to the slightly differing quartz field habitats through long-term isolation by IBE.

An IBT might serve as a strong RI barrier in the central region, where the co-occurring central *O. oviforme* (61, 62 and 72) and *O. nanum* populations formed distinct gene pools. Differences in flowering times were documented by Ihlenfeldt (1978) in this hybridisation zone, with *O. oviforme* flowering four weeks earlier than *O. nanum*. This could cause a mismatch in reproductive times, which has been recorded in numerous species (reviewed by Hendry and Day, 2005).

### Small-scale analyses uncovered introgression and hybridisation events

#### The northern region: an example of isolation by distance.

The regional STRUCTURE analyses uncovered not only a high number of different gene pools but also different genetic structural patterns of the gene pools within each distribution area (Fig. 5C–E). In the northern region, only pure or almost pure gene pools were detected, indicating a strictly limited gene flow between more distant populations. In this area, IBD might be the most important factor for these northern populations.

#### The central region: the secret of the hybridisation zone uncovered.

In the central region, which extends only ~20 km in length, where both species co-exist (Ihlenfeldt, 1978, 2017), the three populations (61, 62 and 72) resembled *O. oviforme* morphologically, while the surrounding quartz field habitats were occupied by *O. nanum*. These central *O. oviforme* populations did not possess any of the western *O. oviforme* gene pools (Fig. 5B, D). Instead, they were hybrids of *O. nanum* gene pools either from the central and the northern gene pool (population 61 and 62) or from three different central gene pools (population 72). Here, one (population 61 and 62) or even two (population 72) introgression events must have occurred. An even distribution of the gene pools within each central *Oophytum* population indicates hybridisation and backcrossing events over a longer period of time (Runemark *et al.*, 2019). In summary, all three central populations characterised morphologically as *O. oviforme* were genetically only hybrids of *O. nanum*.

The hybridisation zone is characterised by a high density of quartz fields (U. Schmiedel, personal observations), which could enable successful dispersal of the seeds or capsules over medium to long distances by water and wind to overcome the strong RI barrier between island populations. In subsequent isolation, the emerged hybrids might have backcrossed with each other, resulting in these now visible stable hybrid gene pools. These quartz fields are particularly diverse in their habitat characteristics (Schmiedel and Jürgens, 1999; Schmiedel, 2002), forcing an evolution of novel variations by adaptation during backcrossing (Abbott *et al.*, 2013; Runemark *et al.*, 2019). Thus, the morphological similarity between the central hybrid populations and the western *O. oviforme* putatively developed convergently.

Hybridisation is a common phenomenon in plant evolution. According to Mallet (2005), ≥25 % of plants and 10 % of animals evolved through hybridisation and introgression events. Species are often incompletely isolated and can evolve in different directions, before they re-establish contact for gene exchange (Mallet, 2005). These introgressive hybridisations could cause the observed contradictions between phylogenetic and taxonomic trees in *Oophytum*. This would explain the conflict in our phylogenetic analyses, where all central *O. oviforme* hybrids clustered as a subclade within *O. nanum* and the taxonomic classification of these populations resulted in central ‘*O. oviforme*’.

#### *The western region: a snapshot of an ongoing introgression within* O. oviforme.

The populations of western *O. oviforme* consisted of four gene pools (Fig. 5E). In contrast to the hybrids in the central region, introgressions occurred in varying amounts, ranging from neglectable in both outer populations (63 and 76) to about one-quarter in the two core populations. Also, the composition of these gene pools varied in comparison of the central stable hybrid populations, with only a few individuals within a population admixed and others pure. According to Runemark *et al.* (2019), this indicates an ongoing hybridisation process. Hence, the hybridisation process in western *O. oviforme* seems to have occurred in a more recent past, and the introgression of the invasive gene pool into a stable population gene pool is not yet complete.

### (Re-)spreading of O. nanum

According to the ‘core–periphery’ hypothesis (Brown, 1984), edge populations of plants and animals are genetically impoverished due to diverse deteriorating biotic and/or abiotic conditions. This leads to less fitness and density of individuals accompanied by an asymmetrical gene flow (summarised by Duncan *et al.*, 2015). Diverse plant and animal taxa do not correspond to this ‘core–periphery’ rule, which is based on ecological and geographical factors (Duncan *et al.*, 2015). This also applied to our taxa: within the marginal populations of *O. nanum*, the northernmost edge population (51) and the eastern populations (50, 72 and 75) had the highest diversity rates within the whole *Oophytum* metapopulation.

If taxa do not correspond to the ‘core–periphery’ rule, alternative factors must enhance genetic diversity. Several historical or evolutionary processes could contribute to this phenomenon (Xu *et al.*, 2017; Carvalho *et al.*, 2019; Lawrence *et al.*, 2020). Southern Africa has experienced major climatic changes in the past (Midgley *et al.*, 2010; Dupont *et al.*, 2011), which have had far-reaching impacts on flora and fauna. The timespan of palaeoclimatic changes triggering the genomic evolution of *Oophytum* can be traced back only by an indirect estimation. Klak *et al.* (2004) published an astonishing high lineage diversification within the Ruschioideae of 0.53–1.32 Mya^−1^ based on molecular clock analyses of cpDNA. Given that our haplotype analyses were also based on cpDNA, we counted the haplotypes of each evolutionary line starting at the unknown common *Oophytum* ancestor and found two to four evolutionary steps within both *O. nanum* and *O. oviforme*. Taking a mean lineage diversification of 0.92 Mya, *Oophytum* might have evolved ~2.8 Mya (1.9–3.7 Mya). This rough estimate would date the starting point of the evolutionary period of *Oophytum* back to the transition of late Pliocene to the Pleistocene recurring ice ages, with their periodical climate changes. During these periods, the competing Fynbos and Succulent Karoo Biomes contracted and expanded each in north and south directions (Midgley and Roberts, 2001). During glaciation epochs, southern Africa was more humid, and the Fynbos vegetation covered large parts of the current Succulent Karoo (Midgley *et al.*, 2010). Arid-adapted taxa might have retreated and survived in refugia with harsh arid and edaphically special habitats (Midgley *et al.*, 2010), such as quartz fields. Within these isolated refugia, adaptations to differences in these habitats might have occurred. During interglacial epochs, diverged plants of the Succulent Karoo re-expanded (Midgley *et al.*, 2010; Dupont *et al.*, 2011) and hybridised at recontacts of diverged refugial gene pools. The edge populations 51 in the north and 50 in the east with a tripartite gene pool could represent such refugial hybrids (Fig. 5B). This is also supported by the studies by Petit *et al.* (2003) and by Hampe and Petit (2005), who detected that edge populations exhibited a higher genetic diversity compared with internal populations, which play an important role for the survival of taxa. During past climate fluctuations, edge populations might have survived challenging conditions at single marginal locations (Petit *et al.*, 2003).

However, we have also identified hybrids with two to three gene pools in the central hybridisation zone: *O. oviforme* populations 61, 62 and 72 and *O. nanum* populations 67 and 75. Repeated migration into refugia, isolated diversification and re-expansion of *O. nanum* during periodic glaciation and interglaciation phases make these hybrid genomes with stable gene pool proportions plausible.

### Implications for conservation management in the Knersvlakte

The phylogenetic and phylogeographical patterns of the locally endemic and habitat-specialised genus *Oophytum* clearly highlight the regional differentiation into genetically distinct populations, as reported earlier for other Knersvlakte taxa (e.g. Ellis *et al.*, 2006, 2007; Musker *et al.*, 2020; Boucher *et al.*, 2024) and suggest limited genetic exchange among the populations across the Knersvlakte. These results have strong implications for conservation management. A consequence is the irreplaceability of the plant populations of different parts of the Knersvlakte. A decline or even a complete extinction of populations in one of the regions would result in the complete loss of distinct gene pools, to a further reduction of the already low genetic diversity within the metapopulation, hence to a reduced ability of the species to adapt to changing environmental conditions (Lande and Shannon, 1996).

Activities such as prospecting and mining (Adhikari *et al.*, 2021), infrastructure development (Lucrezi *et al.*, 2014), illegal harvesting of succulent plants for international trading (Sajeva *et al.*, 2007; Goettsch *et al.*, 2015) and unsustainable grazing and trampling (Van Tonder *et al.*, 2014) can result in the extirpation of local populations, which, in the case of *Oophytum*, might lead to the complete extinction of a distinct and irreplaceable gene pool or even of an entire species (Hufford and Mazer, 2003). The genetic distinctiveness of the local populations would also have implications for the future transplanting (or sowing of seeds) of poached plants that have been seized and are currently cultivated in large numbers (Frantz *et al.*, 2022). As a consequence, the highly heterogeneous gene pool in the Knersvlakte can only be protected effectively in its entirety. Particularly, areas with highly distinct gene pools, such as the south-west of the Knersvlakte or the refugia areas in the south-east and upper north, should be given special attention in nature conservation planning and management.

## Conclusions

The combination of phylogenetic, phylogeographical and ecological analyses of *Oophytum*, including palaeoclimatic aspects, revealed an illuminating snapshot of a complex genus evolution that exemplifies the extremely fast radiation within Ruschioideae. We could resolve the problematic relationship within this small, endemic and highly specialised genus. Its metapopulation split into three main gene pools early on, reflecting their geographical distribution rather than taxonomic units. Our findings suggest that the western species, *O. oviforme*, evolved via the probable relic ‘*O. nordenstamii*’ population. Colonisation of the slightly more humid western quartz fields was accompanied by a gene pool loss that could be interpreted as a founder effect. The speciation of the sister species, *O. nanum*, in the slightly more arid regions of the Knersvlakte appears to have occurred via different processes. This species has two main gene pools, putatively a primary northern and a derivative central–east gene pool. A new species description of the northern group (under the suggested name ‘*Oophytum ihlenfeldtianum*’) would be required.

Periodic Pleistocene glaciations might have caused repeated retreats into northern and south-eastern refugia, differentiation in isolation and hybridisation events during re-spreading. These processes resulted in the present genetic structure of hybrid gene pools in the central zone and refugial edge populations. We could show that the populations in the central hybrid zone, morphologically resembling ‘*O. oviforme*’, are genetically *O. nanum* hybrids that have evolved convergently to their sister species.

We were also able to trace back a range of different adaptation strategies of *Oophytum* to survive as a dwarf, compact plant in a small and slowly but permanently changing arid distribution area of ~2500 km^2^. Adaptations to a mosaic of small quartz islands differing in their habitat conditions, in combination with a strongly restricted gene flow, resulted in a splintering of the three main gene pools into diverse small subunits, each with low genetic diversity. We identified diverse differentiation processes of *Oophytum* populations at different sites of the distribution area. The evolution of the island populations appears to be an interplay between different RI barriers, such as IBD or IBE. Occasionally, these barriers have been surpassed by migrations via medium- and long-distance dispersal, in combination with founder effects, hybridisation events in the past and ongoing introgressions.

Our results provide important information needed for conservation management decisions of the herein studied taxa. Each island gene pool of the metapopulation is unique and fragile. Anthropogenic impact and uninformed conservation attempts, e.g. genetic exchange through transplanting or reseeding, might have unpredictable consequences for species development and richness in this hotspot of diversity and endemism of the arid Succulent Karoo Biome.

## SUPPLEMENTARY DATA

Supplementary data are available at *Annals of Botany* online and consist of the following.

Table S1: Taxa list and NCBI accession numbers.

Table S2: Chloroplast primer list and AFLP adapter and primer.

Table S3: PCR ingredients.

Table S4: PCR conditions.

Table S5: Population genetic structure of 18 *Oophytum* populations.

Table S6: Results of a one-way PERMANOVA*.*

Table S7: Frequency result of GenAlEx: band patterns for 18 *Oophytum* populations.

Table S8: GenAlEx result: band frequency for 18 *Oophytum* populations.

Figure S1. (A–C) Bayesian inference (BI) 50 % majority rule consensus trees. (D–F) Maximum parsimony (MP) 50 % majority rule consensus trees. (G–I) Maximum likelihood (ML) 50 % majority rule consensus trees.

Figure S2: Splitstree v.5 network of the four chloroplast marker alignment.

Figure S3: STRUCTURE HARVESTER: result of all 18 populations.

Figure S4: STRUCTURE (CLUMPP-DISTRUCT): results of all 18 *Oophytum* populations.

Figure S5: STRUCTURE (CLUMPP-DISTRUCT): results of the single analyses for the *Oophytum* populations of each of the three main regions.

Figure S6: STRUCTURE HARVESTER: result of six northern populations.

Figure S7: STRUCTURE HARVESTER: result of six central and two eastern populations.

Figure S8: STRUCTURE HARVESTER: result of four western populations.

Figure S9: Bayesian inference 50 % majority rule consensus tree of the 18 *Oophytum* population AFLP-Dataset.

Figure S10: Splitstree v.5 network of the 18 *Oophytum* population AFLP-Dataset.

Figure S11: Graphical result of a GenAlEx AMOVA.

Figure S12: Result of the GenAlEx Mantel-Test.

mcae207_suppl_Supplementary_Materials

## Notes

In memory of Professor Dr Hans-Dieter Ihlenfeldt (1932–2023).
