# Supplementary material for: Diversity on a small scale: phylogeography of the locally endemic dwarf succulent genus Oophytum (Aizoaceae) in the Knersvlakte of South Africa
Source: Ann Bot. 2024 Dec 4;135(4):735–56. doi: 10.1093/aob/mcae207 (PMC11904907; doi:10.1093/aob/mcae207)
Supplement: mcae207_suppl_Supplementary_Materials [file mcae207_suppl_supplementary_materials.pdf]

# TABLES

**Table S1:** Taxa list & NCBI Accession Numbers

| Taxon | Collection number | Collection date | Collector | District | Country | Abbr. phylogeny | Group | NCBI Accession Number (matK) | NCBI Accession Number (trnL-trnF) | NCBI Accession Number (trnQ-rps16) | NCBI Accession Number (trnS-trnG) | AFLP | PCA |
|---|---|---|---|---|---|---|---|---|---|---|---|---|---|
| Oophytum nanum | 124838_1-10 | 5.11.2011 | Ute Schmiedel | Western Cape | South Africa | Ona38_1_N | north | OM776891 | OM728401 | OM728431 | OM728461 | ✓ | - |
| Oophytum nanum | 124839_1-10 | 6.11.2011 | Ute Schmiedel | Western Cape | South Africa | | north | - | - | - | - | - | - |
| Oophytum nanum | 124840_1-10 | 15.11.2011 | Ute Schmiedel | Western Cape | South Africa | Ona40_2_C | centre | OM776892 | OM728402 | OM728432 | OM728462 | - | - |
| Oophytum oviforme | 124841_1-10 | 18.11.2011 | Ute Schmiedel | Western Cape | South Africa | Oov41_1_W | west | OM776908 | OM728418 | OM728448 | OM728478 | - | - |
| Oophytum oviforme | 124842_1-10 | 21.11.2011 | Ute Schmiedel | Western Cape | South Africa | Oov42_1_C | centre | OM776909 | OM728419 | OM728449 | OM728479 | - | - |
| Oophytum nanum | 124843_1-10 | 21.11.2011 | Ute Schmiedel | Western Cape | South Africa | | centre | - | - | - | - | - | - |
| Oophytum nanum | 124850_1-10 | 13.4.2012 | Ute Schmiedel | Western Cape | South Africa | Ona50_1_E | east | OM776893 | OM728403 | OM728433 | OM728463 | ✓ | ✓ |
| Oophytum nanum | 124851_1-10 | 14.4.2012 | Ute Schmiedel | Western Cape | South Africa | Ona51_7_N | north | OM776894 | OM728404 | OM728434 | OM728464 | ✓ | ✓ |
| Oophytum nanum | 124852_1-10 | 14.4.2012 | Ute Schmiedel | Western Cape | South Africa | Ona52_1_N | north | OM776895 | OM728405 | OM728435 | OM728465 | - | ✓ |
| Oophytum nanum | 124853_1-10 | 14.4.2012 | Ute Schmiedel | Western Cape | South Africa | | north | - | - | - | - | - | ✓ |
| Oophytum nanum | 124854_1-10 | 14.4.2012 | Ute Schmiedel | Western Cape | South Africa | Ona54_1_N | north | OM776896 | OM728406 | OM728436 | OM728466 | ✓ | ✓ |
| Oophytum nanum | 124855_1-10 | 14.4.2012 | Ute Schmiedel | Western Cape | South Africa | Ona55_4_N | north | OM776897 | OM728407 | OM728437 | OM728467 | ✓ | ✓ |
| Oophytum nanum | 124856_1-10 | 14.4.2012 | Ute Schmiedel | Western Cape | South Africa | Ona56_9_C | centre | OM776898 | OM728408 | OM728438 | OM728468 | ✓ | ✓ |
| Oophytum oviforme | 124857_1-10 | 15.4.2012 | Ute Schmiedel | Western Cape | South Africa | Oov57_2_C | centre | OM776910 | OM728420 | OM728450 | OM728480 | - | ✓ |
| Oophytum nanum | 124858_1-10 | 15.4.2012 | Anna-Lena Rau | Western Cape | South Africa | | centre | - | - | - | - | - | ✓ |
| Oophytum nanum | 124859_1-10 | 15.4.2012 | Anna-Lena Rau | Western Cape | South Africa | Ona59_1_C | centre | OM776899 | OM728409 | OM728439 | OM728469 | ✓ | ✓ |
| Oophytum nanum | 124860_1-10 | 15.4.2012 | Anna-Lena Rau | Western Cape | South Africa | Ona60_4_C | centre | OM776900 | OM728410 | OM728440 | OM728470 | - | ✓ |
| Oophytum oviforme | 124861_1-10 | 15.4.2012 | Anna-Lena Rau | Western Cape | South Africa | Oov61_1_C | centre | OM776911 | OM728421 | OM728451 | OM728481 | ✓ | ✓ |

| Taxon | Collection number | Collection date | Collector | District | Country | Abbr. phylogeny | Group | NCBI Accession Number (matK) | NCBI Accession Number (trnL-trnF) | NCBI Accession Number (trnQ-rps16) | NCBI Accession Number (trnS-trnG) | AFLP | PCA |
|---|---|---|---|---|---|---|---|---|---|---|---|---|---|
| Oophytum oviforme | 124862_1-10 | 15.4.2012 | Anna-Lena Rau | Western Cape | South Africa | Oov62_1_C | centre | OM776912 | OM728422 | OM728452 | OM728482 | ✓ | ✓ |
| Oophytum oviforme "nordenstamii" | 124863_1-10 | 15.4.2012 | Anna-Lena Rau | Western Cape | South Africa | Ono63_2_W | west | OM776907 | OM728417 | OM728447 | OM728477 | ✓ | ✓ |
| Oophytum nanum | 124864_1-10 | 16.4.2012 | Anna-Lena Rau | Western Cape | South Africa | Ona64_8_N | north | OM776901 | OM728411 | OM728441 | OM728471 | - | ✓ |
| Oophytum nanum | 124865_1-10 | 16.4.2012 | Anna-Lena Rau | Western Cape | South Africa | | north | - | - | - | - | - | ✓ |
| Oophytum nanum | 124866_1-10 | 17.4.2012 | Anna-Lena Rau | Western Cape | South Africa | Ona66_3_C | centre | OM776902 | OM728412 | OM728442 | OM728472 | - | ✓ |
| Oophytum nanum | 124867_1-10 | 17.4.2012 | Anna-Lena Rau | Western Cape | South Africa | Ona67_9_C | centre | OM776903 | OM728413 | OM728443 | OM728473 | ✓ | ✓ |
| Oophytum nanum | 124868_1-10 | 19.4.2012 | Anna-Lena Rau | Western Cape | South Africa | Ona68_8_C | centre | OM776904 | OM728414 | OM728444 | OM728474 | ✓ | ✓ |
| Oophytum nanum | 124869_1-10 | 19.4.2012 | Anna-Lena Rau | Western Cape | South Africa | | centre | - | - | - | - | - | ✓ |
| Oophytum nanum | 124870_1-10 | 19.4.2012 | Anna-Lena Rau | Western Cape | South Africa | | centre | - | - | - | - | - | ✓ |
| Oophytum oviforme | 124871_1-10 | 20.4.2012 | Anna-Lena Rau | Western Cape | South Africa | Oov71_1_W | west | OM776913 | OM728423 | OM728453 | OM728483 | - | ✓ |
| Oophytum oviforme | 124872_1-10 | 20.4.2012 | Anna-Lena Rau | Western Cape | South Africa | Oov72_1_C | centre | OM776914 | OM728424 | OM728454 | OM728484 | ✓ | ✓ |
| Oophytum oviforme | 124873_1-10 | 20.4.2012 | Anna-Lena Rau | Western Cape | South Africa | | west | - | - | - | - | - | ✓ |
| Oophytum nanum | 124874_1-10 | 24.4.2012 | Anna-Lena Rau | Western Cape | South Africa | Ona74_5_C | east | OM776905 | OM728415 | OM728445 | OM728475 | - | ✓ |
| Oophytum nanum | 124875_1-10 | 24.4.2012 | Anna-Lena Rau | Western Cape | South Africa | Ona75_1_C | east | OM776906 | OM728416 | OM728446 | OM728476 | ✓ | ✓ |
| Oophytum oviforme | 124876_1-10 | 25.4.2012 | Anna-Lena Rau | Western Cape | South Africa | Oov76_3_W | west | OM776915 | OM728425 | OM728455 | OM728485 | ✓ | ✓ |
| Oophytum oviforme | 124877_1-10 | 25.4.2012 | Anna-Lena Rau | Western Cape | South Africa | Oov77_1_W | west | OM776916 | OM728426 | OM728456 | OM728486 | ✓ | ✓ |
| Oophytum oviforme | 124878_1-10 | 25.4.2012 | Anna-Lena Rau | Western Cape | South Africa | Oov78_1_W | west | OM776917 | OM728427 | OM728457 | OM728487 | ✓ | ✓ |
| Delosperma spec. | 30930 | 17.3.1992 | Heidrun Hartmann | Eastern Cape | South Africa | Delo_spec_H30930 | | OM776888 | OM728398 | OM728428 | OM728458 | | |
| Gibbaeum geminium | 109862 | 14.3.1998 | Ute Schmiedel | Western Cape | South Africa | Gibb_gemi_S109862 | | OM776889 | OM728399 | OM728429 | OM728459 | | |
| Malephora crassa | 8981 | 16.4.1977 | Heidrun Hartmann | Western Cape | South Africa | Male_cras_H8981 | | OM776890 | OM728400 | OM728430 | OM728460 | | |

| Taxon | Collection number | Collection date | Collector | District | Country | Abbr. phylogeny | Group | NCBI Accession Number (matK) | NCBI Accession Number (trnL-trnF) | NCBI Accession Number (trnQ-rps16) | NCBI Accession Number (trnS-trnG) | AFLP | PCA |
|---|---|---|---|---|---|---|---|---|---|---|---|---|---|
| Oophytum nanum | 791 | | C.Klak | | | Ona_Klak791 | | KF132732.1 | KF132838.1 | KF131952.1 | KF133213.1 | | |
| Dicrocaulon brevifolium | 1642 | | C.Klak | | | Dicr_brev_Klak1642 | | KF132676.1 | KF132802.1 | KF131898.1 | KF133158.1 | | |
| Diplosoma retroversum | 835 | | C.Klak | | | Dipl_retr_Klak835 | | KF132679.1 | AJ558071.1 | KF131901.1 | KF133161.1 | | |
| Monilaria moniliformis | 787 | | C.Klak | | | Moni_moni_Klak787 | | KF132722.1 | AJ558074.1 | KF131942.1 | KF133203.1 | | |

**Table S2:** cp Primer list and AFLP adapter & primer

| Region | Primer name | Primer sequence (5´-3´) | Author |
|---|---|---|---|
| *mat*K | *mat*K2.1f | CCTATCCATCTNGAAATCTTNG | Kew Royal Botanic Gardens, 2012 |
| | *mat*K5r | GTTCTAGCACAAGAAAGCCG | |
| *trn*G-*trn*S | *trn*G(UCC)f | GAACGAATCACACTTTTACCA C | Hamilton, 1999 |
| | *trn*S(GCU)r | GCCGCTTTAGTCCACTCAGC | |
| | ***trn*SG-514Faiz** | **CTGGCGAGGTACTGATCAGG** | **this study** |
| | ***trn*SG-552Raiz** | **TATTATTCCCACGGCCTGGC** | |
| *trn*Q(UUG)-*rps*16 5' | *trn*Q(UUG) | TCCTTCCGTCCCAGAGCA | Shaw et al., 2007 |
| | *rps*16 5` | CCTCGAATTTRGAGCCGCT | |
| *trn*L-*trn*F | *trn*L-c | CGAAATCGGTAGACGCTACG | Taberlet et al., 1991 |
| | *trn*L-d | GGGGATAGAGGGACTTGAAC | |
| | *trn*L-e | GGTTCAAGTCCCTCTATCCC | |
| | *trn*F-f | ATTTGAACTGGTGACACGAG | |
| AFLP Adapter | AdEcoO | CTCGTAGACTGCGTACC | Vos et al., 1995 |
| | AdEcoU | AATTGGTACGCAGTCTAC | |
| | AdMseO | GACGATGAGTCCTGAG | |
| | AdMseU | TACTCAGGACTCAT | |
| AFLP pre-amplification | PEco+A | GACTGCGTACCAATTCA | |
| | PMse+A | GATGAGTCCTGAGTAAA | |
| AFLP selective-amplification | SEcoA+TG | GACTGCGTACCAATTCATG | |
| | SMseA+GG | GATGAGTCCTGAGTAAAGG | |

**Table S3:** PCR ingredients

| | *mat*K | *trn*L-*trn*F | *trn*Q-*rps*16 | *trn*G-*trn*S |
|---|---|---|---|---|
| **BSA (bovine serum albumin)** | 0.01 mg/ml | - | 0.01 mg/ml | 0.01 mg/ml |
| **Bio Budget Buffer (B or B2)** | 1.2x Buffer B | 1x Buffer B | 1.2x Buffer B | 1x Buffer A |
| **DNA** | 15 ng/µl | 15 ng/µl | 15 ng/µl | 20 ng/µl |
| **dNTP's** | 0.24 mM | 0.2 mM | 0.24 mM | 0.2 mM |
| **$MgCl_2$** | 3 mM | 3.75 mM | 3 mM | 2.5 mM |
| **Primer (for- & backward)** | 3 µM each | 2 µM each | 3 µM each | 2 µM each |
| **Bio Budget Taq (standard or hot start)** | 0.1 U/µl (standard) | 0.1 U/µl (standard) | 0.1 U/µl (standard) | 0.1 U/µl (hot start) |
| **ad $H_2O$** | 10 µl | 10 µl | 10 µl | 10 µl |

**Table S4:** PCR conditions

| | ***mat*K** | ***trn*L-*trn*F** | ***trn*Q-*rps*16** | ***trn*G-*trn*S** |
|---|---|---|---|---|
| Step 1 (initial denaturation) | 94° C - 60 sec | 95° C - 180 sec | 95° C - 180 sec | 95° C - 900 sec |
| Step 2 (denaturation) | 94° C - 30 sec | 95° C - 45 sec | 95° C - 45 sec | 95° C - 45 sec |
| Step 3 (annealing) | 52° C - 40 sec | 50° C - 45 sec | 60° C - 45 sec | 57° C - 60 sec |
| Step 4 (elongation) | 72° C - 40 sec | 72° C - 60 sec | 72° C - 60 sec | 72° C - 60 sec |
| number of cycles (Step 2 to Step 4) | 35 | 35 | 40 | 40 |
| Step 5 (final elongation) | 72° C - 300 sec | 72° C - 420 sec | 72° C - 420 sec | 72° C - 420 sec |

**Table S5: Population genetic structure of 18 *Oophytum* populations** calculated with AFLPsurv 1.0 for all 18 populations together and for each region separately. Ht: total gene diversity, Hw: the mean gene diversity within populations, Hb: the average gene diversity among populations in excess of that observed within populations (= genetic differentiation among populations), **$F_{ST}$: Wright's fixation index** (measuring the genetic correlation between pairs of genes sampled within a population relative to pairs of genes sampled within the overall set of populations), S.E.: standard error, Var: variance.

| n | Ht | Hw | Hb | $F_{ST}$ |
|---|---|---|---|---|
| **18 (all)** | 0.2295 | 0.1325 | 0.0970 | **0.4225** |
| S.E. | | 0.005695 | 0.002485 | 0.032163 |
| Var | | 0.000032 | 0.000006 | 0.001034 |
| **7 (only centre)** | 0.2089 | 0.1255 | 0.0835 | **0.4013** |
| S.E. | | 0.006129 | 0.012382 | 0.095252 |
| Var | | 0.000038 | 0.000153 | 0.009073 |
| **5 (only north)** | 0.2158 | 0.1473 | 0.0684 | **0.3159** |
| S.E. | | 0.013505 | 0.000000 | 0.040116 |
| Var | | 0.000182 | -0.000014 | 0.001609 |
| **2 (only east)** | 0.2333 | 0.1578 | 0.0755 | **0.3236** |
| S.E. | | 0.002891 | 0.000000 | 0.012392 |
| Var | | 0.000008 | 0.000000 | 0.000154 |
| **4 (only west)** | 0.1731 | 0.1137 | 0.0594 | **0.3418** |
| S.E. | | 0.006713 | 0.000000 | 0.000000 |
| Var | | 0.000045 | -0.000032 | -0.000382 |

**Table S6: Results of a one-way PERMANOVA** (PAST 4.11, similarity index: dice) Bonferroni corrected p-values of the 18 *Oophytum* population AFLP-Dataset, (Permutation N: 9999, Total sum of squares: 19.01, Within-group sum of squares: 3.472, F: 38.18, p (same): 0.0001).
**Not significant values > 0.05 are marked in bold.**

| | **Ona38** | **Ona52** | **Ona54** | **Ona55** | **Ona56** | **Ona59** | **Oov61** | **Oov62** | **Ona67** | **Ona68** | **Oov72** | **Ona75** | **Oov77** | **Oov78** | **Ona50** | **Ona51** | **Ono63** | **Oov76** |
|---|---|---|---|---|---|---|---|---|---|---|---|---|---|---|---|---|---|---|
| **Ona38** | | 0.0153 | 0.0153 | 0.0153 | 0.0153 | 0.0306 | 0.0459 | 0.0306 | 0.0459 | 0.0153 | 0.0153 | 0.0153 | 0.0153 | 0.0306 | 0.0306 | 0.0153 | 0.0153 | 0.0153 |
| **Ona52** | 0.0153 | | 0.0153 | 0.0153 | 0.0153 | 0.0153 | 0.0306 | 0.0153 | 0.0153 | 0.0153 | 0.0306 | 0.0153 | 0.0153 | 0.0153 | 0.0153 | 0.0153 | 0.0153 | 0.0153 |
| **Ona54** | 0.0153 | 0.0153 | | 0.0459 | 0.0153 | 0.0153 | 0.0153 | 0.0153 | 0.0153 | 0.0153 | 0.0306 | 0.0306 | 0.0153 | 0.0153 | 0.0153 | 0.0153 | 0.0153 | 0.0153 |
| **Ona55** | 0.0153 | 0.0153 | 0.0459 | | 0.0153 | 0.0153 | 0.0306 | 0.0153 | 0.0153 | 0.0153 | 0.0153 | 0.0153 | 0.0306 | 0.0153 | 0.0153 | 0.0153 | 0.0153 | 0.0306 |
| **Ona56** | 0.0153 | 0.0153 | 0.0153 | 0.0153 | | 0.0153 | 0.0306 | 0.0306 | 0.0153 | 0.0153 | 0.0153 | 0.0153 | 0.0306 | 0.0153 | 0.0153 | 0.0153 | 0.0306 | 0.0306 |
| **Ona59** | 0.0306 | 0.0153 | 0.0153 | 0.0153 | 0.0153 | | 0.0153 | 0.0153 | 0.0306 | 0.0153 | 0.0153 | 0.0306 | 0.0153 | 0.0153 | 0.0153 | 0.0306 | 0.0306 | 0.0459 |
| **Oov61** | 0.0459 | 0.0306 | 0.0153 | 0.0306 | 0.0306 | 0.0153 | | 0.0153 | 0.0306 | 0.0306 | **0.0612** | 0.0153 | 0.0153 | 0.0306 | 0.0153 | 0.0459 | 0.0153 | 0.0153 |
| **Oov62** | 0.0306 | 0.0153 | 0.0153 | 0.0153 | 0.0306 | 0.0153 | 0.0153 | | 0.0306 | 0.0153 | 0.0153 | 0.0153 | 0.0153 | 0.0306 | 0.0306 | 0.0153 | 0.0153 | 0.0153 |
| **Ona67** | 0.0459 | 0.0153 | 0.0153 | 0.0153 | 0.0153 | 0.0306 | 0.0306 | 0.0306 | | 0.0306 | 0.0306 | 0.0153 | 0.0153 | 0.0459 | 0.0306 | 0.0459 | 0.0306 | 0.0459 |
| **Ona68** | 0.0153 | 0.0153 | 0.0153 | 0.0153 | 0.0153 | 0.0153 | 0.0306 | 0.0153 | 0.0306 | | 0.0153 | 0.0306 | 0.0153 | 0.0153 | 0.0153 | 0.0153 | 0.0153 | 0.0153 |
| **Oov72** | 0.0153 | 0.0306 | 0.0306 | 0.0153 | 0.0153 | 0.0153 | **0.0612** | 0.0153 | 0.0306 | 0.0153 | | 0.0153 | 0.0153 | 0.0153 | 0.0153 | **0.1377** | 0.0153 | 0.0153 |
| **Ona75** | 0.0153 | 0.0153 | 0.0306 | 0.0153 | 0.0153 | 0.0306 | 0.0153 | 0.0153 | 0.0153 | 0.0306 | 0.0153 | | 0.0306 | 0.0153 | 0.0153 | 0.0153 | 0.0153 | 0.0153 |
| **Oov77** | 0.0153 | 0.0153 | 0.0153 | 0.0306 | 0.0306 | 0.0153 | 0.0153 | 0.0153 | 0.0153 | 0.0153 | 0.0153 | 0.0306 | | 0.0153 | 0.0153 | 0.0153 | 0.0306 | 0.0153 |
| **Oov78** | 0.0306 | 0.0153 | 0.0153 | 0.0153 | 0.0153 | 0.0153 | 0.0306 | 0.0306 | 0.0459 | 0.0153 | 0.0153 | 0.0153 | 0.0153 | | 0.0459 | 0.0153 | **0.0612** | 0.0153 |
| **Ona50** | 0.0306 | 0.0153 | 0.0153 | 0.0153 | 0.0153 | 0.0153 | 0.0153 | 0.0306 | 0.0306 | 0.0153 | 0.0153 | 0.0153 | 0.0153 | 0.0459 | | **0.0612** | 0.0153 | 0.0153 |
| **Ona51** | 0.0153 | 0.0153 | 0.0153 | 0.0153 | 0.0153 | 0.0306 | 0.0459 | 0.0153 | 0.0459 | 0.0153 | **0.1377** | 0.0153 | 0.0153 | 0.0153 | 0.0612 | | 0.0153 | 0.0306 |
| **Ono63** | 0.0153 | 0.0153 | 0.0153 | 0.0153 | 0.0306 | 0.0306 | 0.0153 | 0.0153 | 0.0306 | 0.0153 | 0.0153 | 0.0153 | 0.0306 | **0.0612** | 0.0153 | 0.0153 | | 0.0153 |
| **Oov76** | 0.0153 | 0.0153 | 0.0153 | 0.0306 | 0.0306 | 0.0459 | 0.0153 | 0.0153 | 0.0459 | 0.0153 | 0.0153 | 0.0153 | 0.0153 | 0.0153 | 0.0153 | 0.0306 | 0.0153 | |

**Table S7: Frequency-result of GenAlEx:** Band patterns for 18 *Oophytum* populations. No. Bands = No. of different Bands, No. Bands Freq. ≥5% = Number of different Bands with a Frequency ≥ 5%, No. Private Bands = Number of Bands Unique to a Single Population, No. LComm Bands (<=25%) = Number of Locally Common Bands (Freq. >= 5%) Found in 25% or Fewer Populations, No. LComm Bands (<=50%) = Number of Locally Common Bands (Freq. ≥ 5%) found in 50% or fewer Populations, h = Diversity = 1 - (p^2 + q^2), uh = Unbiased Diversity = (N / (N-1)) * h, where for Haploid Binary data, p = Band Freq. and q = 1 - p.

| **Population** | **38** | **51** | **52** | **54** | **55** | **56** | **59** | **61** | **62** | **67** | **68** | **72** | **50** | **75** | **63** | **76** | **77** | **78** |
|---|---|---|---|---|---|---|---|---|---|---|---|---|---|---|---|---|---|---|
| **No. Bands** | 115 | 146 | 110 | 113 | 112 | 116 | 117 | 114 | 117 | 125 | 111 | 112 | 134 | 137 | 87 | 113 | 101 | 95 |
| **No. Bands Freq. >= 5%** | 115 | 146 | 110 | 113 | 112 | 116 | 117 | 114 | 117 | 125 | 111 | 112 | 134 | 137 | 87 | 113 | 101 | 95 |
| **No. Private Bands** | 2 | 5 | 1 | 0 | 1 | 0 | 1 | 1 | 1 | 1 | 1 | 1 | 1 | 3 | 1 | 2 | 1 | 0 |
| **No. LComm Bands (<=25%)** | 7 | 22 | 8 | 12 | 7 | 15 | 15 | 1 | 8 | 16 | 9 | 12 | 14 | 9 | 5 | 14 | 9 | 5 |
| **No. LComm Bands (<=50%)** | 44 | 67 | 43 | 42 | 44 | 46 | 49 | 38 | 42 | 48 | 35 | 42 | 54 | 54 | 26 | 41 | 37 | 35 |
| **Mean h** | **0.114** | **0.163** | 0.097 | 0.100 | 0.092 | 0.090 | 0.079 | 0.099 | 0.097 | 0.097 | 0.072 | 0.069 | **0.111** | **0.118** | 0.074 | 0.084 | 0.088 | 0.090 |
| **SE of Mean h** | 0.011 | 0.012 | 0.010 | 0.010 | 0.010 | 0.010 | 0..008 | 0.010 | 0.010 | 0.010 | 0.009 | 0008 | 0.010 | 0.011 | 0.009 | 0.009 | 0.010 | 0.010 |
| **Mean uh** | **0.130** | **0.189** | 0.109 | 0.112 | 0.103 | 0.102 | 0.089 | 0.117 | 0.110 | 0.113 | 0.081 | 0.080 | **0.129** | **0.133** | 0.084 | 0.097 | 0.098 | 0.103 |
| **SE of Mean uh** | 0.012 | 0.014 | 0.011 | 0.012 | 0.011 | 0.011 | 0.009 | 0.012 | 0.011 | 0.012 | 0.010 | 0.010 | 0.012 | 0.012 | 0.010 | 0.011 | 0.011 | 0.011 |
| | **north** | | | | | **centre** | | | | | | | **east** | | **west** | | | |

**Table S8: GenAlEx-Result:** Band Frequency for 18 *Oophytum* populations, Allele Frequency (p & q), Samples Size, No. Alleles, No. Effective Alleles, Information Index, Diversity, Unbiased Diversity and Percentage of Polymorphic Loci. N = Number of Samples, Na = Number of Different Alleles, Ne = Number of Effective Alleles = 1 / (p^2 + q^2), I = Shannon's Information Index = -1* (p * Ln (p) + q * Ln(q)), h = Diversity = 1 - (p^2 + q^2), uh = Unbiased Diversity = (N / (N-1)) * h, Where for Haploid Binary data, p = Band Freq. and q = 1 - p.

| Pop | | N | Na | Ne | I | h | uh | Percentage of Polymorphic Loci |
|---|---|---|---|---|---|---|---|---|
| **38** | **Mean** | 8.746 | 0.773 | 1.191 | **0.173** | **0.114** | 0.130 | 33.71% |
| | **SE** | 0.046 | 0.057 | 0.020 | 0.016 | 0.011 | 0.012 | |
| **51** | **Mean** | 7.761 | 1.027 | 1.272 | **0.247** | **0.163** | 0.189 | 47.35% |
| | **SE** | 0.041 | 0.059 | 0.021 | 0.017 | 0.012 | 0.014 | |
| **52** | **Mean** | 9.697 | 0.708 | 1.159 | 0.147 | 0.097 | 0.109 | 29.17% |
| | **SE** | 0.048 | 0.055 | 0.018 | 0.015 | 0.010 | 0.011 | |
| **54** | **Mean** | 9.807 | 0.716 | 1.167 | 0.151 | 0.100 | 0.112 | 28.79% |
| | **SE** | 0.035 | 0.054 | 0.019 | 0.015 | 0.010 | 0.012 | |
| **55** | **Mean** | 9.693 | 0.708 | 1.150 | 0.140 | 0.092 | 0.103 | 28.41% |
| | **SE** | 0.055 | 0.054 | 0.017 | 0.015 | 0.010 | 0.011 | |
| **56** | **Mean** | 9.727 | 0.723 | 1.147 | 0.139 | 0.090 | 0.102 | 28.41% |
| | **SE** | 0.044 | 0.054 | 0.017 | 0.014 | 0.010 | 0.011 | |
| **59** | **Mean** | 9.814 | 0.735 | 1.120 | 0.127 | 0.079 | 0.089 | 29.17% |
| | **SE** | 0.038 | 0.054 | 0.014 | 0.013 | 0.008 | 0.009 | |
| **61** | **Mean** | 6.822 | 0.716 | 1.162 | 0.150 | 0.099 | 0.117 | 28.41% |
| | **SE** | 0.031 | 0.054 | 0.018 | 0.015 | 0.010 | 0.012 | |
| **62** | **Mean** | 8.814 | 0.754 | 1.155 | 0.150 | 0.097 | 0.110 | 31.06% |
| | **SE** | 0.032 | 0.055 | 0.017 | 0.015 | 0.010 | 0.011 | |
| **67** | **Mean** | 7.803 | 0.769 | 1.158 | 0.149 | 0.097 | 0.113 | 29.55% |
| | **SE** | 0.038 | 0.054 | 0.018 | 0.015 | 0.010 | 0.012 | |
| **68** | **Mean** | 9.902 | 0.655 | 1.113 | 0.112 | 0.072 | 0.081 | 23.48% |
| | **SE** | 0.023 | 0.051 | 0.015 | 0.013 | 0.009 | 0.010 | |
| **72** | **Mean** | 7.811 | 0.648 | 1.108 | 0.107 | 0.069 | 0.080 | 22.35% |
| | **SE** | 0.039 | 0.051 | 0.014 | 0.013 | 0.008 | 0.010 | |
| **50** | **Mean** | 7.735 | 0.837 | 1.179 | **0.170** | **0.111** | 0.129 | 32.95% |
| | **SE** | 0.040 | 0.055 | 0.018 | 0.015 | 0.010 | 0.012 | |
| **75** | **Mean** | 9.742 | 0.894 | 1.195 | **0.182** | **0.118** | 0.133 | 37.50% |
| | **SE** | 0.047 | 0.057 | 0.019 | 0.016 | 0.011 | 0.012 | |
| **63** | **Mean** | 8.924 | 0.576 | 1.117 | 0.116 | 0.074 | 0.084 | 24.62% |
| | **SE** | 0.020 | 0.053 | 0.015 | 0.013 | 0.009 | 0.010 | |
| **76** | **Mean** | 7.867 | 0.693 | 1.134 | 0.130 | 0.084 | 0.097 | 26.52% |
| | **SE** | 0.031 | 0.053 | 0.016 | 0.014 | 0.009 | 0.011 | |
| **77** | **Mean** | 9.837 | 0.670 | 1.143 | 0.136 | 0.088 | 0.098 | 28.79% |
| | **SE** | 0.031 | 0.055 | 0.017 | 0.014 | 0.010 | 0.011 | |
| **78** | **Mean** | 8.769 | 0.644 | 1.145 | 0.139 | 0.090 | 0.103 | 28.41% |
| | **SE** | 0.038 | 0.055 | 0.017 | 0.014 | 0.010 | 0.011 | |
| **Total** | **Mean** | 8.848 | 0.736 | 1.156 | 0.148 | 0.096 | 0.110 | 29.92% |
| | **SE** | 0.017 | 0.013 | 0.004 | 0.003 | 0.002 | 0.003 | 1.33% |

# FIGURES

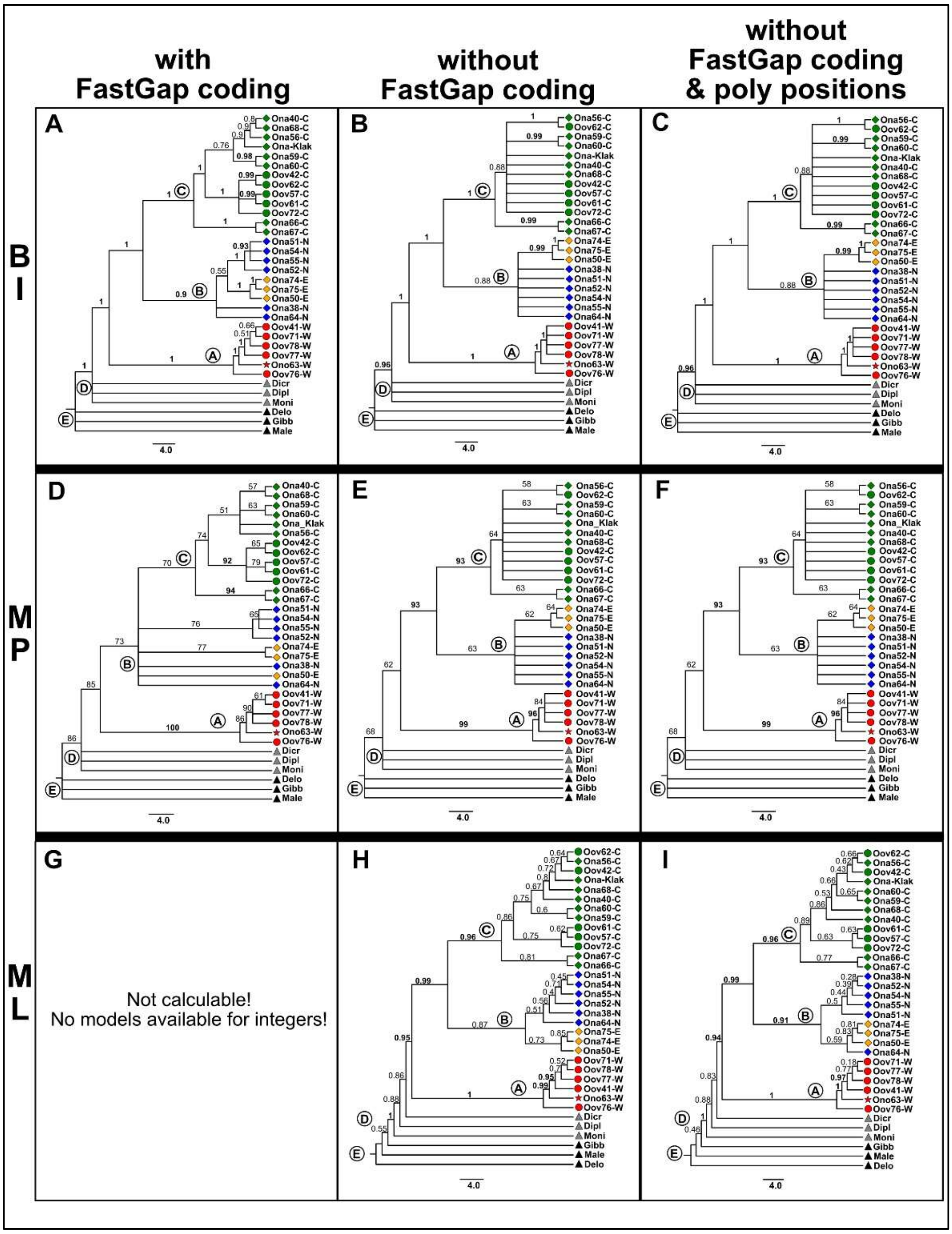


**Fig. S1 A-C: Bayesian Inference (BI)** 50% majority rule consensus trees (numbers above lines are posterior probability values), **A** with FastGap coding, **B** without FastGap coding, **C** without FastGap coding and variable poly positions; **D-F: Maximum Parsimony (MP)** 50% majority rule consensus trees (numbers above lines are bootstrap values), **D** with FastGap coding, **E** without FastGap coding, **F** without FastGap coding and variable poly positions; **G-H: Maximum Likelihood (ML)** 50% majority rule consensus trees (numbers above lines posterior probability values), **G** a calculation with FastGap coding was not possible, **H** without FastGap coding (numbers above lines posterior probability values), **I** without FastGap coding and variable poly positions (numbers above lines posterior probability values), (blue diamonds = northern group of *O. nanum*, green diamonds = central group of *O. nanum*, orange diamonds = eastern group of *O. nanum*, green circles = central group of *O. oviforme*, red circles = western group of *O. oviforme*, red star = "*O. nordenstamii*" population). All trees are ladderized (increasing order) and proportional.

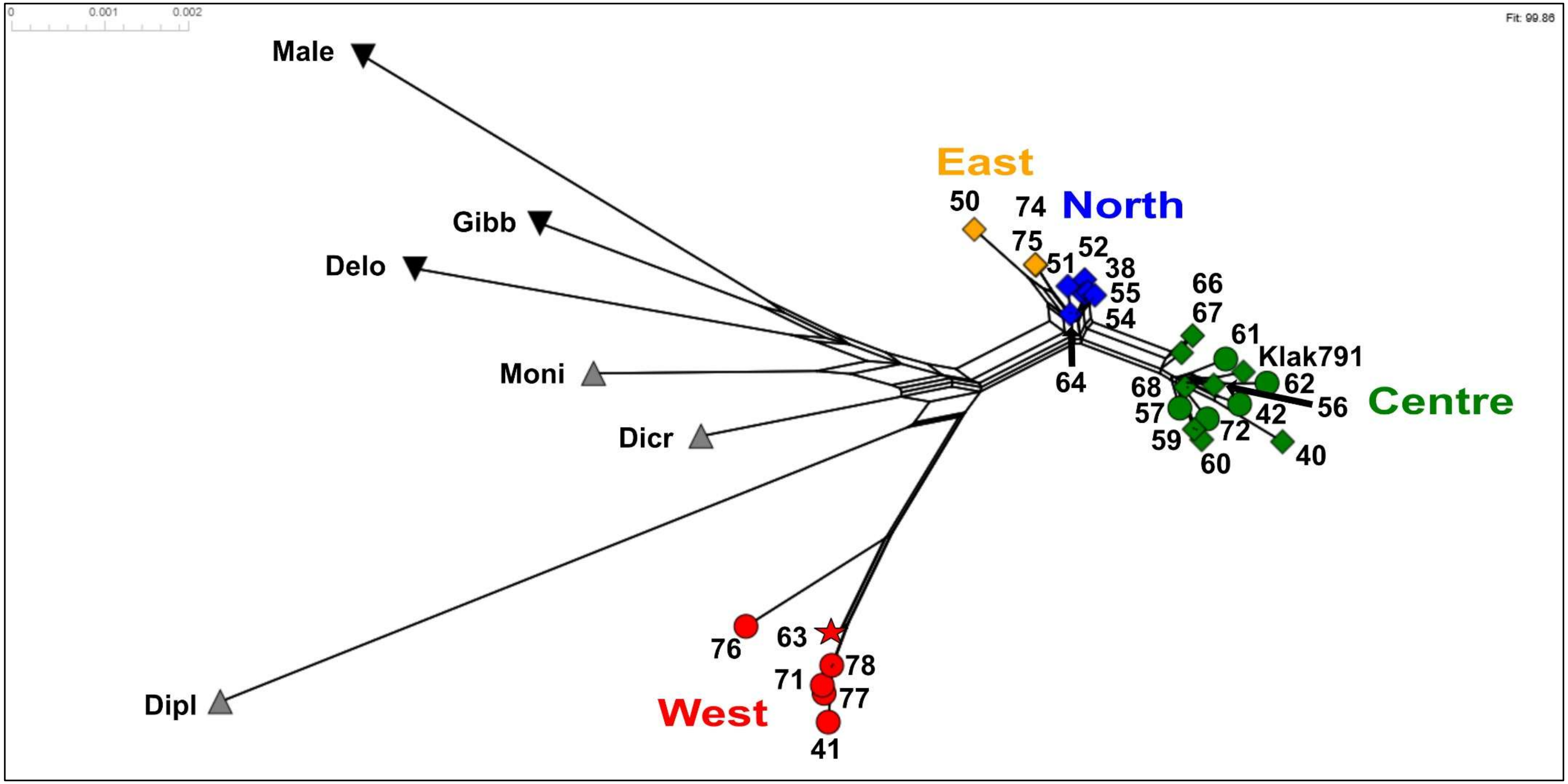


**Fig. S2: Splitstree5-Network of the four cp-Marker Alignment** with 34 Samples (28 *Oophytum* samples & 3 *Dicrocaulon*-Clade samples & 3 outgroup samples). Numbers represent the population number, blue diamond = northern *O. nanum* populations, orange diamond = eastern O. *nanum* populations, green diamond = central *O. nanum* populations, green circles = central *O. oviforme* populations, red circles = western *O. oviforme* populations, red star = former "*O. nordenstamii*" population, Delo = *Delosperma spec*., Dicr = *Dicrocaulon brevifolium*, Dipl = *Diplosoma retroversum*, Gibb = *Gibbaeum geminum*, Male = *Malephora crassa*, Moni = *Monilaria moniliformis*.

| # K | Reps | Mean LnP(K) | Stdev LnP(K) | Ln'(K) | \|Ln''(K)\| | Delta K |
|---|---|---|---|---|---|---|
| 1 | 20 | -15941.7000 | 0.8820 | NA | NA | NA |
| 2 | 20 | -14570.5100 | 27.2958 | 1371.190000 | 60.535000 | 2.217742 |
| **3** | 20 | -13259.8550 | 6.4718 | 1310.655000 | 595.335000 | **91.988525** |
| 4 | 20 | -12544.5350 | 49.4600 | 715.320000 | 58.520000 | 1.183178 |
| 5 | 20 | -11887.7350 | 45.2738 | 656.800000 | 208.015000 | 4.594600 |
| 6 | 20 | -11438.9500 | 65.6511 | 448.785000 | 37.615000 | 0.572953 |
| 7 | 20 | -11027.7800 | 97.2146 | 411.170000 | 32.780000 | 0.337192 |
| 8 | 20 | -10583.8300 | 109.5525 | 443.950000 | 81.645000 | 0.745259 |
| 9 | 20 | -10221.5250 | 115.7022 | 362.305000 | 31.080000 | 0.268621 |
| 10 | 20 | -9828.1400 | 119.6375 | 393.385000 | 186.905000 | 1.562261 |
| 11 | 20 | -9621.6600 | 434.0789 | 206.480000 | 211.820000 | 0.487976 |
| 12 | 20 | -9203.3600 | 134.1829 | 418.300000 | 1518.865000 | 11.319367 |
| 13 | 20 | -10303.9250 | 3199.6921 | -1100.565000 | 0.600000 | 0.000188 |
| 14 | 20 | -11403.8900 | 4672.4728 | -1099.965000 | 2335.390000 | 0.499819 |
| 15 | 20 | -10168.4650 | 2647.1504 | 1235.425000 | 536.640000 | 0.202724 |
| 16 | 20 | -9469.6800 | 2648.8884 | 698.785000 | 360.085000 | 0.135938 |
| 17 | 20 | -8410.8100 | 190.2151 | 1058.870000 | 1164.980000 | 6.124539 |
| 18 | 20 | -8516.9200 | 374.6116 | -106.110000 | 73.095000 | 0.195122 |
| 19 | 20 | -8549.9350 | 391.6330 | -33.015000 | NA | NA |

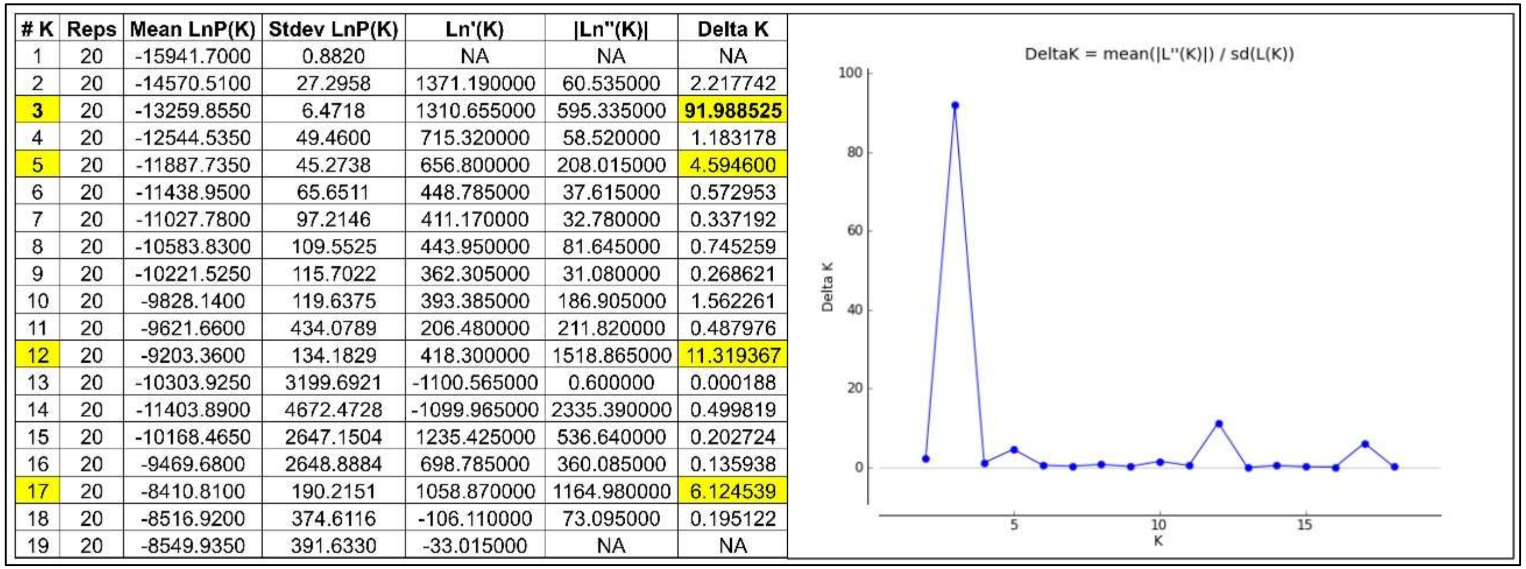


**Fig. S3: STRUCTURE HARVESTER-Result of all 18 populations** (overall analysis): EvannoTable & DeltaK

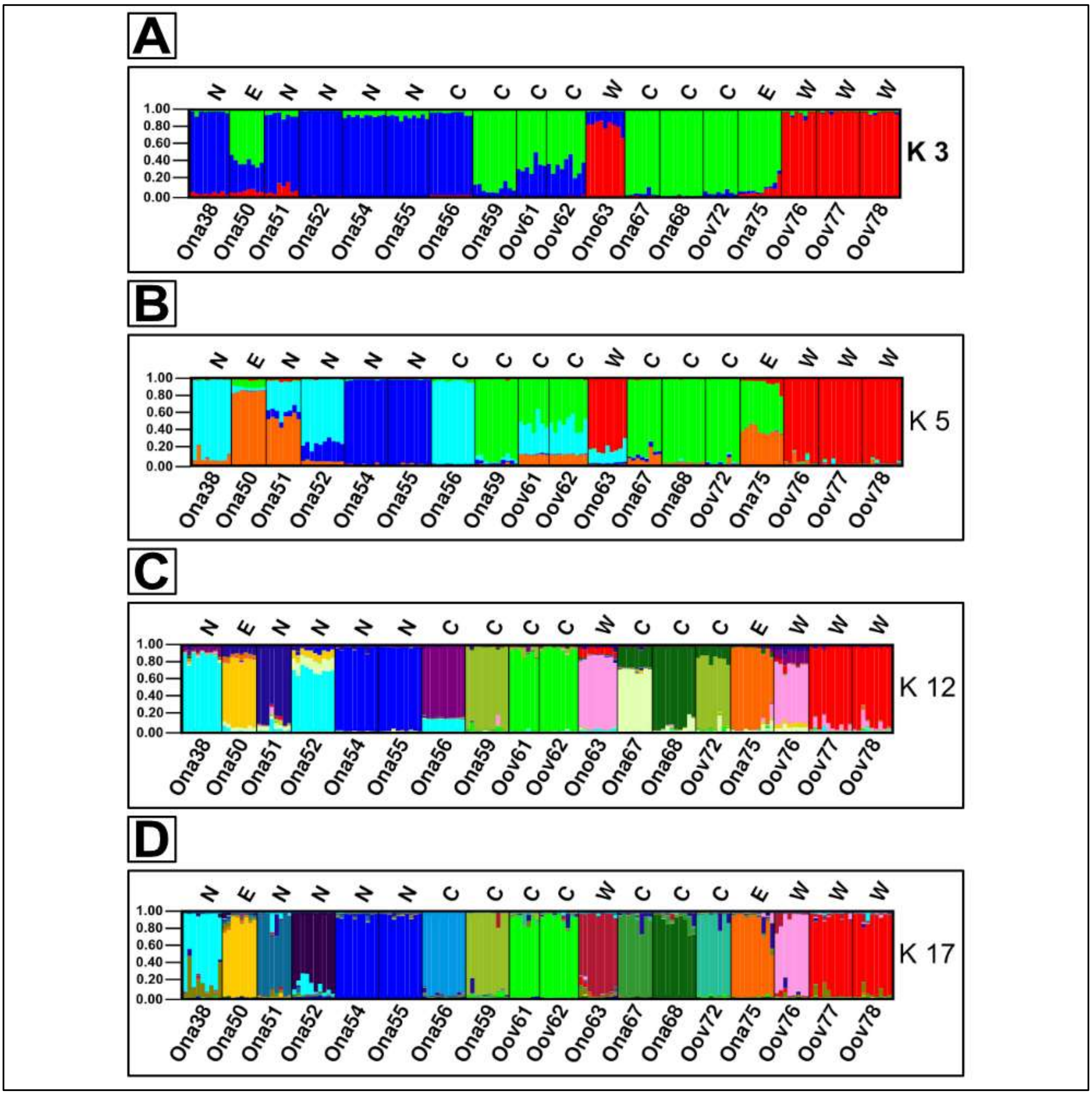


**Fig. S4: STRUCTURE (CLUMPP-DISTRUCT)-Results of all 18 *Oophytum* populations**. **A:** barplot for the best ΔK 3, **B:** for ΔK 5, **C:** for ΔK 12, **D:** for ΔK 17.

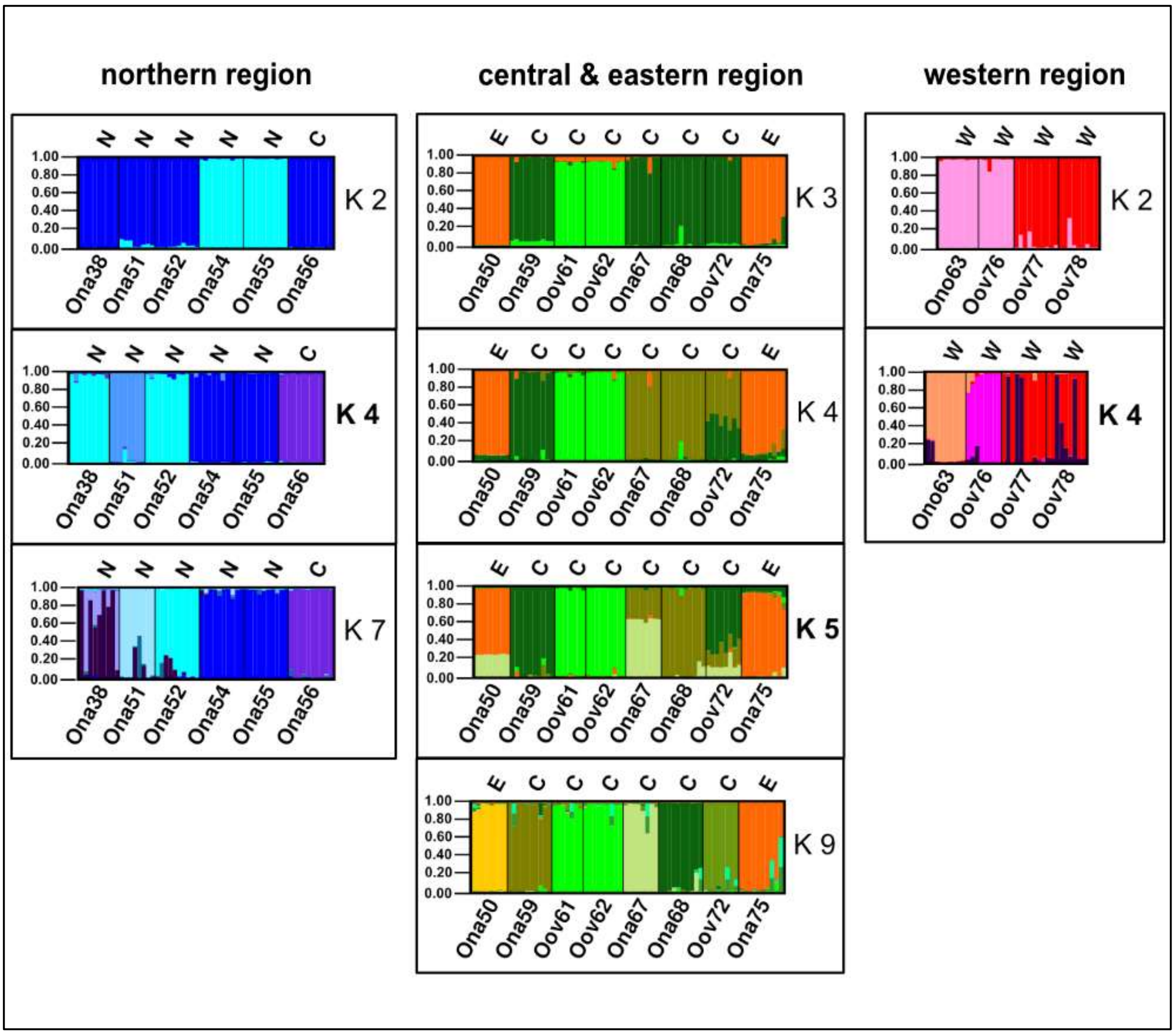


**Fig. S5: STRUCTURE (CLUMPP-DISTRUCT)-Results of the single analyses for the *Oophytum* populations of each of the three main regions (north, central-east and west).** Barplots for all with Structure Harvester identified ΔK values are shown for each region. The highest ΔK value for each analysis is marked in bold (northern region K 4, central & eastern region K 5 and western region K 4).

| # K | Reps | Mean LnP(K) | Stdev LnP(K) | Ln'(K) | \|Ln''(K)\| | Delta K |
|---|---|---|---|---|---|---|
| 1 | 20 | -4965.0700 | 1.1323 | NA | NA | NA |
| 2 | 20 | -4310.4950 | 2.1476 | 654.575000 | 167.225000 | 77.866892 |
| 3 | 20 | -3823.1450 | 35.0393 | 487.350000 | 46.660000 | 1.331649 |
| **4** | 20 | -3382.4550 | 2.9226 | 440.690000 | 365.225000 | **124.966063** |
| 5 | 20 | -3306.9900 | 839.4797 | 75.465000 | 307.155000 | 0.365887 |
| 6 | 20 | -3538.6800 | 236.0182 | -231.690000 | 696.395000 | 2.950599 |
| 7 | 20 | -3073.9750 | 16.5301 | 464.705000 | 629.220000 | 38.065188 |
| 8 | 20 | -3238.4900 | 687.2748 | -164.515000 | NA | NA |

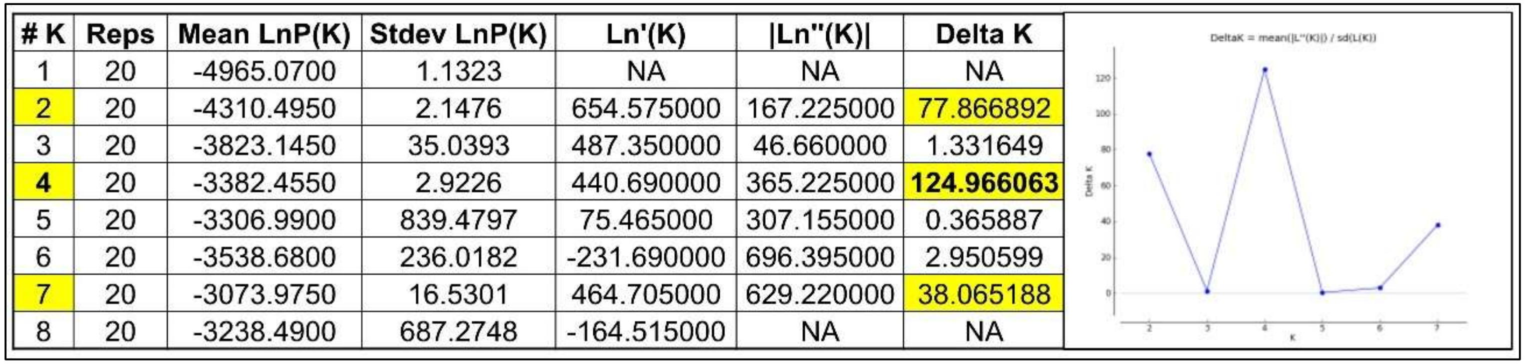


**Fig. S6: STRUCTURE HARVESTER-Result of 6 northern populations** (single analysis): EvannoTable & DeltaK

| # K | Reps | Mean LnP(K) | Stdev LnP(K) | Ln'(K) | \|Ln''(K)\| | Delta K |
|---|---|---|---|---|---|---|
| 1 | 20 | -5919.9350 | 1.6781 | NA | NA | NA |
| 2 | 20 | -5337.4300 | 9.6376 | 582.505000 | 7.970000 | 0.826970 |
| 3 | 20 | -4746.9550 | 39.8836 | 590.475000 | 161.620000 | 4.052296 |
| 4 | 20 | -4318.1000 | 36.2069 | 428.855000 | 137.370000 | 3.794026 |
| **5** | 20 | -4026.6150 | 43.9384 | 291.485000 | 618.550000 | **14.077665** |
| 6 | 20 | -4353.6800 | 2222.6395 | -327.065000 | 643.475000 | 0.289509 |
| 7 | 20 | -4037.2700 | 1355.9845 | 316.410000 | 18.230000 | 0.013444 |
| 8 | 20 | -3739.0900 | 1372.7037 | 298.180000 | 91.030000 | 0.066314 |
| 9 | 20 | -3531.9400 | 34.1356 | 207.150000 | 223.495000 | 6.547264 |
| 10 | 20 | -3548.2850 | 36.2812 | -16.345000 | NA | NA |

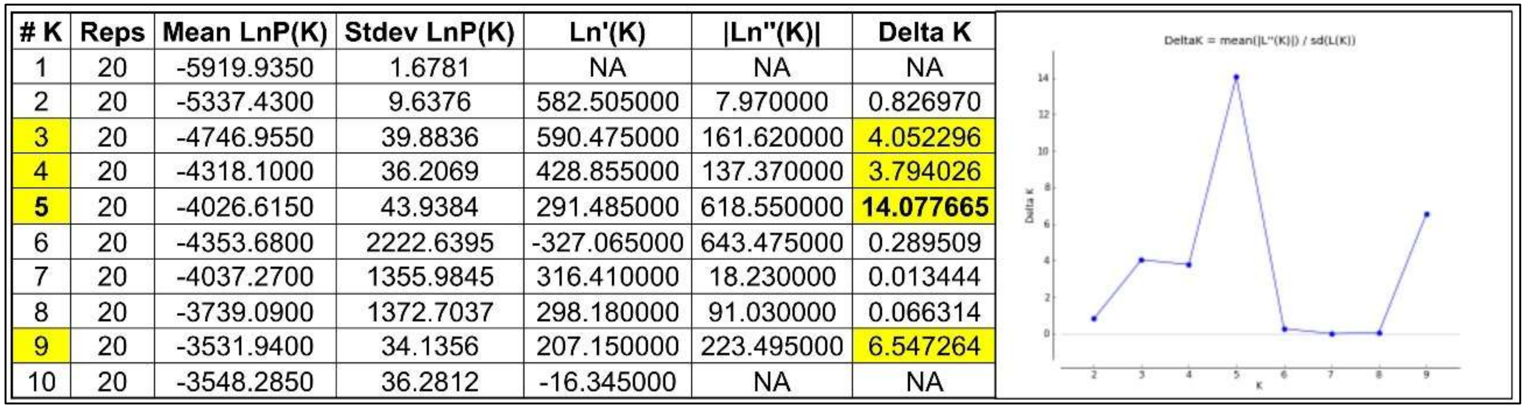


**Fig. S7: STRUCTURE HARVESTER-Result of 6 central & 2 eastern populations** (single analysis): EvannoTable & DeltaK

| # K | Reps | Mean LnP(K) | Stdev LnP(K) | Ln'(K) | \|Ln''(K)\| | Delta K |
|---|---|---|---|---|---|---|
| 1 | 20 | -2389.9800 | 1.4827 | NA | NA | NA |
| 2 | 20 | -1971.7100 | 0.8149 | 418.270000 | 64.200000 | 78.780107 |
| 3 | 20 | -1617.6400 | 60.2926 | 354.070000 | 235.070000 | 3.898817 |
| 4 | 20 | -1498.6400 | 1.7901 | 119.000000 | 124.340000 | **69.457926** |
| 5 | 20 | -1503.9800 | 20.9808 | -5.340000 | 136.545000 | 6.508091 |
| 6 | 20 | -1645.8650 | 391.6991 | -141.885000 | NA | NA |

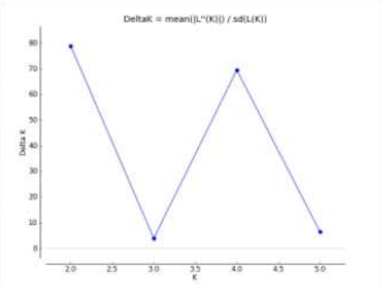


**Fig. S8: STRUCTURE HARVESTER-Result of 4 western populations** (single analysis): EvannoTable & DeltaK

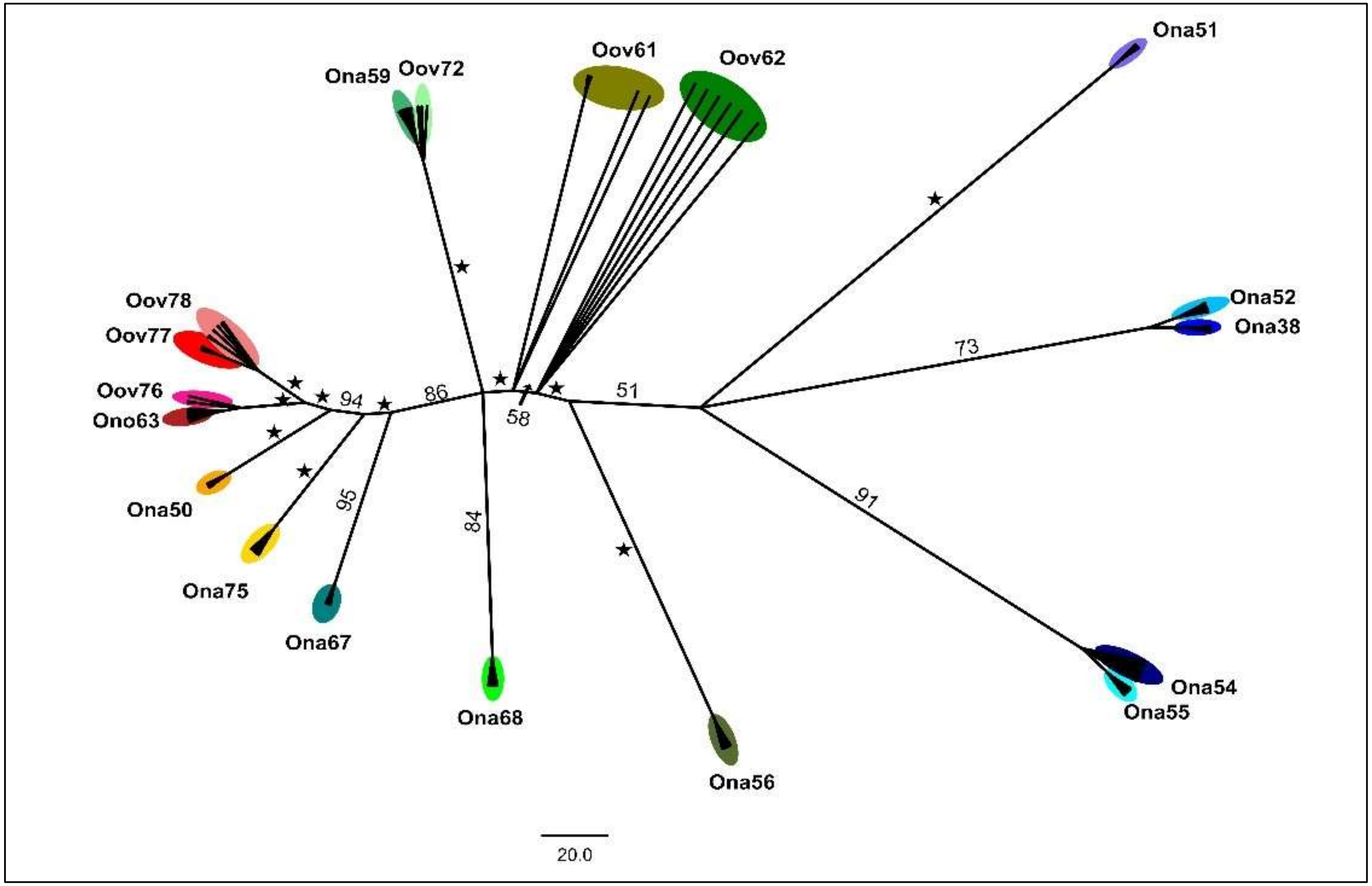


**Fig. S9: Bayesian Inference majority rule consensus tree** of the 18 *Oophytum* population AFLP-Dataset conducted with MrBayes (5 Million replicates, posterior probability values in percent, star = 98-100%). Tree is unrooted and proportional.

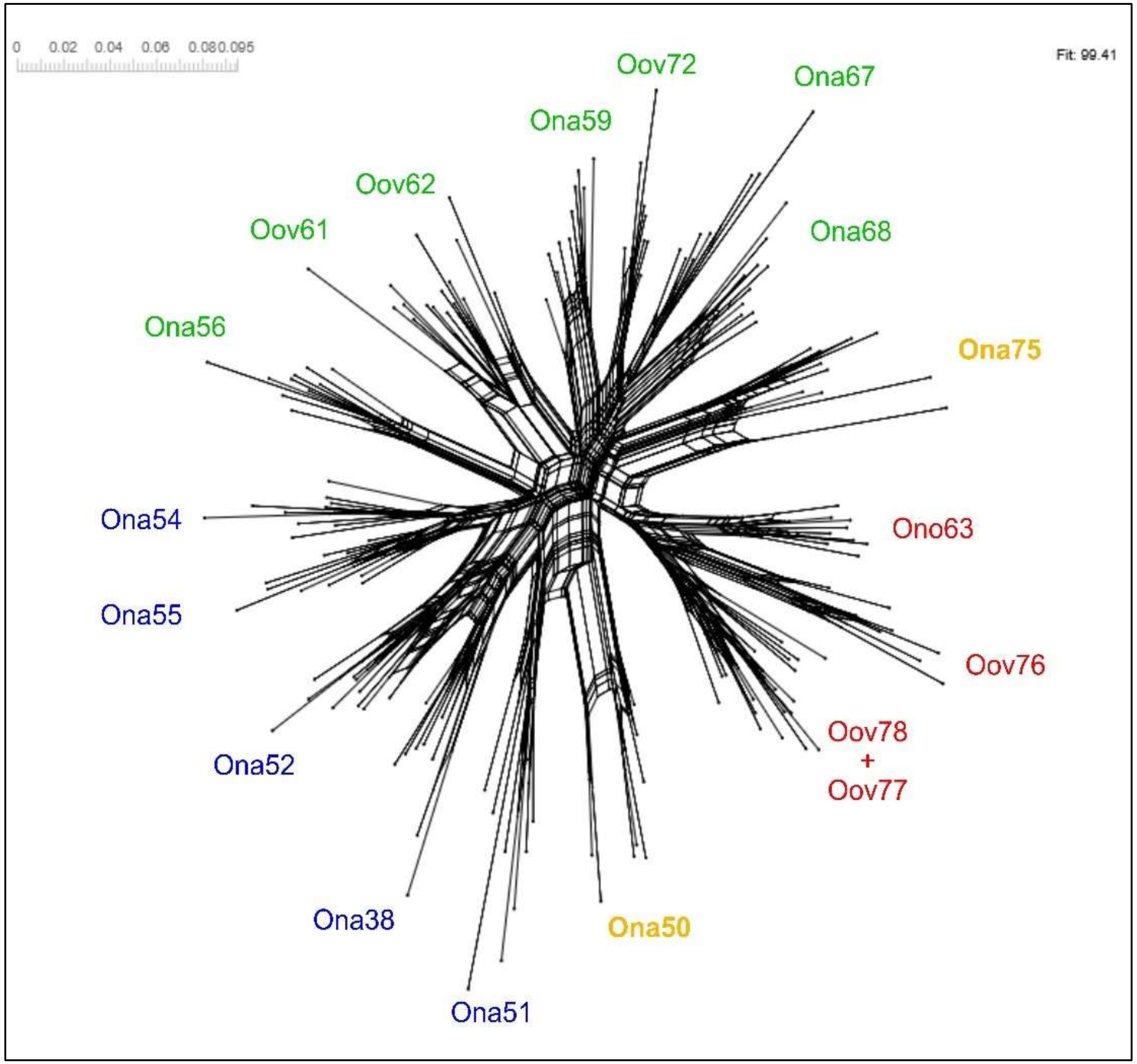


**Fig. S10: Splitstree5-network (hamming distance)** of the 18 *Oophytum* population AFLP-Dataset.

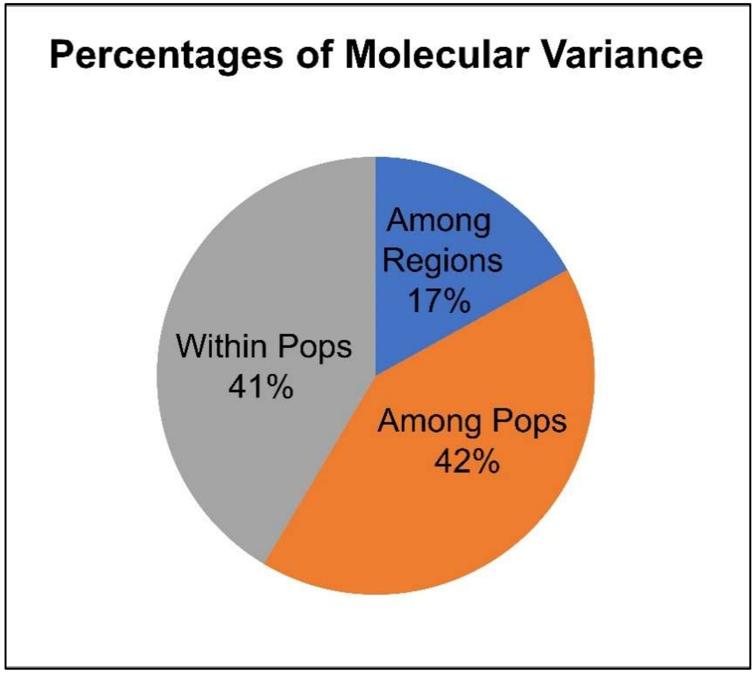


**Fig. S11**: **Graphic result of a GenAlEx AMOVA analysis** based on AFLPdata of 18 *Oophytum* populations with a grouping into four (geographic) regions: west, centre, east and north**,** see Table 4 for statistics.

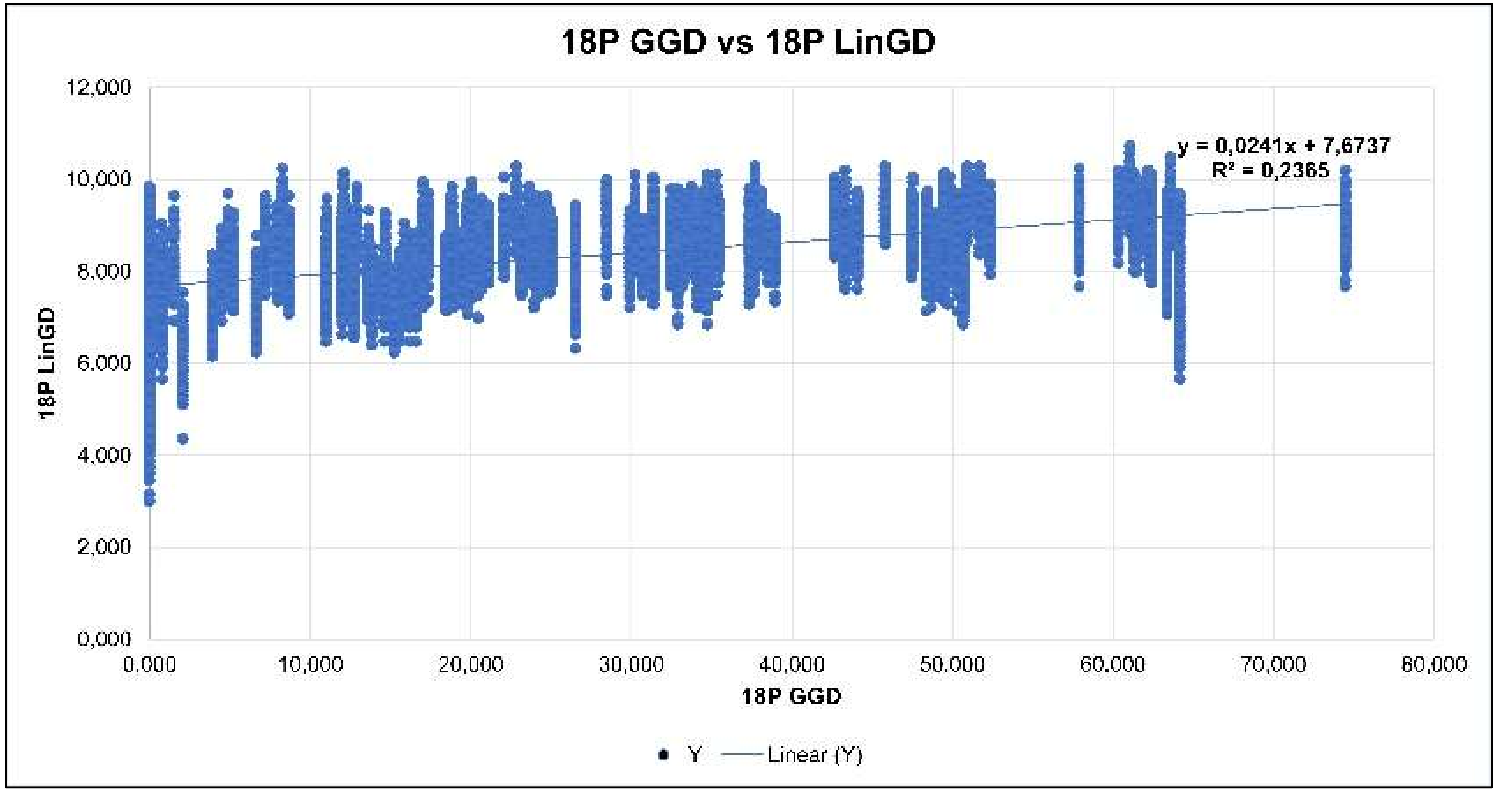


**Fig. S12: Result of the GenAlEx Mantel-Test.** The linear genetic distance (LinGD) of the 18 *Oophytum* Population AFLP-Dataset was plotted in a coordinate system against the geographic genetic distance (GGD).
